# Spatial and Temporal Scales of Range Expansion in Wild *Phaseolus vulgaris*

**DOI:** 10.1093/molbev/msx273

**Published:** 2017-10-23

**Authors:** Andrea Ariani, Jorge Carlos Berny Mier y Teran, Paul Gepts

**Affiliations:** Department of Plant Sciences, Section of Crop and Ecosystem Sciences, University of California, Davis, CA

**Keywords:** climate adaptation, coalescent analysis, crop wild relatives, genotyping‐by‐sequencing, long‐distance dispersal

## Abstract

The wild progenitor of common-bean has an exceptionally large distribution from northern Mexico to northwestern Argentina, unusual among crop wild progenitors. This research sought to document major events of range expansion that led to this distribution and associated environmental changes. Through the use of genotyping-by-sequencing (∼20,000 SNPs) and geographic information systems applied to a sample of 246 accessions of wild *Phaseolus vulgaris*, including 157 genotypes of the Mesoamerican, 77 of the southern Andean, and 12 of the Northern Peru–Ecuador gene pools, we identified five geographically distinct subpopulations. Three of these subpopulations belong to the Mesoamerican gene pool (Northern and Central Mexico, Oaxaca, and Southern Mexico, Central America and northern South America) and one each to the Northern Peru–Ecuador (PhI) and the southern Andean gene pools. The five subpopulations were distributed in different floristic provinces of the Neotropical seasonally dry forest and showed distinct distributions for temperature and rainfall resulting in decreased local potential evapotranspiration (PhI and southern Andes groups) compared with the two Mexican groups. Three of these subpopulations represent long-distance dispersal events from Mesoamerica into Northern Peru–Ecuador, southern Andes, and Central America and Colombia, in chronological order. Of particular note is that the dispersal to Northern Peru–Ecuador markedly predates the dispersal to the southern Andes (∼400 vs. ∼100 ky), consistent with the ancestral nature of the phaseolin seed protein and chloroplast sequences observed in the PhI group. Seed dispersal in common bean can be, therefore, described at different spatial and temporal scales, from localized, annual seed shattering to long‐distance, evolutionarily rare migration.

## Introduction

Common bean (*Phaseolus vulgaris*) is one of the most important crops for human nutrition, being a source of proteins, vitamins, fibers, and essential micronutrients worldwide (Gepts et al. 2008). This species belongs to a highly diversified genus that comprises approximately 70 different species, of which five of them have been domesticated (Freytag and Debouck 2002). Current knowledge highlights the presence of two distinct wild gene pools in common bean, in Mesoamerica and the southern Andes, which were domesticated independently (Gepts 1998; Kwak and Gepts 2009; Kwak et al. 2009; Bitocchi et al. 2013; Schmutz et al. 2014; Vlasova et al. 2016). These domesticated pools underwent local adaptations and diversified into landraces with distinct characteristics (Singh et al. 1991; Chacón et al. 2007). The presence of two gene pools has been identified both in domesticated and wild germplasms with several markers including seed proteins (Gepts et al. 1986), allozymes (Koenig and Gepts 1989), and several types of DNA markers (Chacón et al. 2007; Kwak and Gepts 2009; Bitocchi et al. 2012, 2013; Schmutz et al. 2014). A third gene pool, from Northern Peru and Ecuador, only consists of wild populations (Gepts et al. 1986; Debouck et al. 1993). This gene pool is characterized by a specific phaseolin seed protein type (“I” phaseolin), which is ancestral based on the absence of tandem direct repeats in the phaseolin genes; the type I phaseolin is absent in the Mesoamerican and Andean gene pools (Kami et al. 1995).

Wild common bean has a wide geographical distribution, from northern Mexico to northwestern Argentina, with specific patterns of geographical distribution and population size depending on the different gene pools (Debouck et al. 1993; Freytag and Debouck 2002; Chacón et al. 2007). The Mesoamerican gene pool is distributed in Mexico, Central America, Colombia, and Venezuela, the Intermediate (or Northern Peru–Ecuador) is located in North Peru–Ecuador on the western side of the Andes, and the Andean gene pool on the eastern side of the Andes in southern Peru, Bolivia, and Argentina. Previous analysis of genetic diversity and phylogeographic analysis showed that the wild Mesoamerican gene pool is the one with the broadest geographical distribution and highest nucleotide diversity compared with the other two pools (Chacón et al. 2007; Kwak and Gepts 2009; Bitocchi et al. 2013; Mamidi et al. 2013; Schmutz et al. 2014). These studies also showed a strong predomestication bottleneck in the Andean gene pool that drastically reduced its nucleotide diversity, as well as low nucleotide diversity in the Northern Peru–Ecuador populations.

Due to the complex population structure and evolutionary history of this species, several studies have focused on understanding its origins, development, and evolution in the Americas (Gepts et al. 1986; Koenig and Gepts 1989; Gepts 1998; Chacón et al. 2007; Kwak and Gepts 2009; Bitocchi et al. 2012, 2013; Mamidi et al. 2013; Bellucci et al. 2014; Schmutz et al. 2014; Vlasova et al. 2016). Currently, there are two main hypotheses about the origin of common bean species based on different experimental evidence: the Mesoamerican and the Northern Peru–Ecuador hypothesis. The Mesoamerican hypothesis suggests a Mesoamerican origin of common bean, with the Northern Peru–Ecuador and Andean groups created from two different migrations to South America. This hypothesis is supported by the observation that the majority of *Phaseolus* wild species are located in Mesoamerica (Delgado-Salinas et al. 1999; Freytag and Debouck 2002) and by the higher genetic diversity of the wild Mesoamerican gene pool compared with the other wild populations (Gepts et al. 1986; Koenig and Gepts 1989; Kwak and Gepts 2009; Bitocchi et al. 2013; Mamidi et al. 2013; Schmutz et al. 2014). This hypothesis is also supported by a combined sequence analysis of five genetic loci in the common bean genome and spatial interpolation of population membership (Bitocchi et al. 2012). The Northern Peru–Ecuador hypothesis suggests that common bean originated in the western slopes of the Andes in North Peru–Ecuador, and migrated North to Colombia, Central America, and Mexico, and South to South Peru, Bolivia, and Argentina. This hypothesis is supported by the presence of the ancestral type I phaseolin gene that does not contains tandem direct repeats in its sequence (Kami et al. 1995) and by their chloroplast DNA haplotype that closely resemble the putatively ancestral one (Delgado-Salinas et al. 1999; Chacón et al. 2007).

From its center of origin, wild common bean has been able to spread across the Americas and colonize apparently different environmental niches with different annual rainfall regimes such as dry areas in North Mexico, tropical and humid environments in Central and South America, as well as relatively cold areas on the mountain slopes of the Andes (Cortés et al. 2013). During these colonizations events, wild common bean differentiated into distinct genetic groups (Kwak and Gepts 2009; Bitocchi et al. 2012; Blair et al. 2012). However, it is not clear if these genetic groups have been the results of random demographic processes, such as drift and founder effect, and/or the direct effect of adaptation to new environments.

Understanding the ecological distribution, genetic diversity, population structure, origins, and evolution of a species, in particular of a crop wild relative, is crucial for efficient conservation and management strategies of germplasm collections (Ford-Lloyd et al. 2011). For these reasons, we characterized the genome-wide genetic diversity, population structure, phylogeny, and evolutionary history of a wild common bean panel representative of the eco-geographical distribution of this species. We also integrated the genetic diversity data with the ecological distribution of the individuals genotyped to gain a better understanding about the spread and potential adaptation of this species across the Americas.

## Results

### SNPs and Genetic Diversity Analysis

After retaining only the wild *P. vulgaris* genotypes and applying the final filtering parameters, we obtained 19,126 loci in 246 genotypes representative of the different wild gene pools of common bean. These included 157 genotypes of the Mesoamerican, 77 of the Andean, and 12 of the Northern Peru–Ecuador gene pools (supplementary table S1, Supplementary Material online). SNP density closely resembled gene distribution in the *P. vulgaris* genome (supplementary fig. S1, Supplementary Material online) and was positively correlated with both chromosome length (*r* = 0.85, *P* < 0.001) and gene density (*r* = 0.74 *P < *0.001). Nucleotide diversity (*π*), Tajima’s *D*, linkage blocks, and population differentiation (*F*_st_) were analyzed for the entire sample and for three wild gene pools of common bean (table 1). Among the three gene pools, the Mesoamerican population showed the highest nucleotide diversity (*π *= 1.42e^−5^), twice that of the Andean gene pool (*π = *6.46e^−6^), and only slightly higher (27%) than the Northern Peru–Ecuador gene pool (*π *= 1.05e^−5^). Regarding Tajima’s *D*, the Mesoamerican, and Northern Peru–Ecuador gene pools show positive values, *D* = 1.58 and 0.28, respectively. In contrast, the Andean gene pool showed a negative value (*D* = −0.45).

**Table 1.** Population Genetic Statistics for the SNPs Identified in This Study in the Different Gene Pools of *Phaseolus vulgaris.*

Population	*π*	*D*	Hap (N50)^a^	*F*_st_ PhI	Fst AW	Fst MW
All	1.41e-5	1.52	1,331 (275.86)	NA	NA	NA
MW	1.42e-5	1.52	951 (246.31)	0.30	0.34	0.00
AW	6.46e-6	−0.45	245 (921.04)	0.62	0.00	0.34
PhI	1.04e-5	0.28	27 (693.71)	0.00	0.62	0.30

Note.—All, all the genotypes; MW, Mesoamerican wild; AW, Andean wild; PhI, Northern Peru–Ecuador; *π*, nucleotide diversity; *D*, Tajima’s *D* statistics; *F*_st_, population differentiation statistic.

a Number of haplotype blocks and N50 length.

The Mesoamerican gene pool showed the highest number of haplotype blocks (951), with the shortest median (N50) length (246 kb). The Andean gene pool showed fewer haplotype blocks (245), but the highest N50 (921 kb). The Northern Peru–Ecuador population showed the lowest number of haplotype blocks (27) and an intermediate N50 length (694 kb) compared with the Mesoamerican and Andean groups. Population differentiation analysis (*F*_st_) showed a strong differentiation between the three populations, with the highest value observed between the Northern Peru–Ecuador and Andean populations (*F*_st_= ∼0.6), and the lowest between Northern Peru–Ecuador and Mesoamerican gene pools (*F*_st_ = ∼0.3). The *F*_st_ value between Andean and Mesoamerican gene pools (*F*_st_ = ∼0.34) confirmed previous estimations using whole genome sequence analysis of these populations (Schmutz et al. 2014).

### Molecular Principal Component Analysis, Population Structure, and Phylogenetic Analysis

**Fig. 1.**
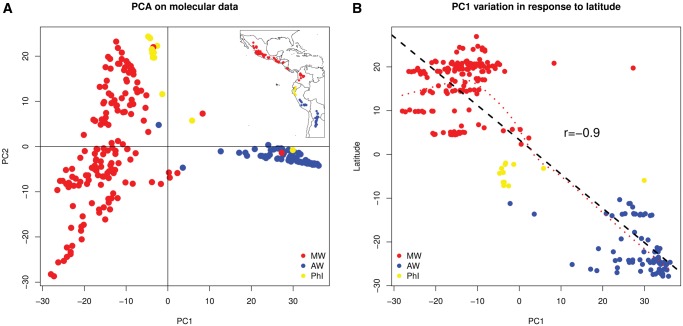
(*A*) Molecular PCA analysis of genetic diversity in wild *Phaseolus vulgaris.* Inset: Geographical distribution of the genotypes analyzed. (*B*) Correlation between molecular PC1 and latitude of wild *P. vulgaris.* Black dashed line: linear correlation; red dotted line: local polynomial regression. Pearson’s correlation coefficient (*r*) is shown. MW, Mesoamerican wild; AW, Andean wild; PhI, Northern Peru–Ecuador.

Molecular principal component analysis (PCA) was performed for the 246 *P. vulgaris* genotypes at the 19,126 loci using the adegenet package implemented in the R environment. The PCA analysis showed that the first principal component (PC1) discriminated mainly between the Mesoamerican/Northern Peru–Ecuador groups and the Andean gene pool (fig. 1*A*), following a latitude-dependent distribution (fig. 1*A* inset). The second principal component (PC2) discriminated mainly between the Northern Peru–Ecuador and Andean gene pools, both showing less to no variability. On the other hand, the Mesoamerican gene pool showed the highest variability on PC2 (fig. 1*A*), suggesting a possible latitudinal cline of genetic differentiation across this gene pool from northern Mexico to Colombia. Linear correlation between PC1 and latitude showed a significant (*P < *2e^−16^) negative correlation (*r* = −0.9) between these variables (fig. 1*B*, black dashed line), whereas local polynomial regression (red dotted lines) had a bimodal distribution, showing independence between these two variables in the Mesoamerican gene pool, and following an negative, almost linear correlation at lower latitudes.

**Fig. 2.**
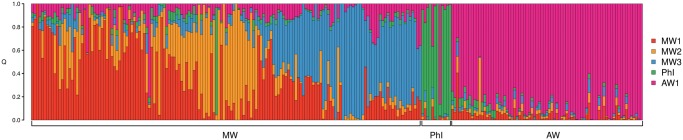
Spatial population clustering and ancestry coefficients estimated with TESS3 using the best number of subpopulations (*K* = 5). The genotypes are sorted by latitude from Northern Mexico to Northwestern Argentina. MW, Mesoamerican wild; AW, Andean wild; PhI, Northern Peru-Ecuador.

**Fig. 3.**
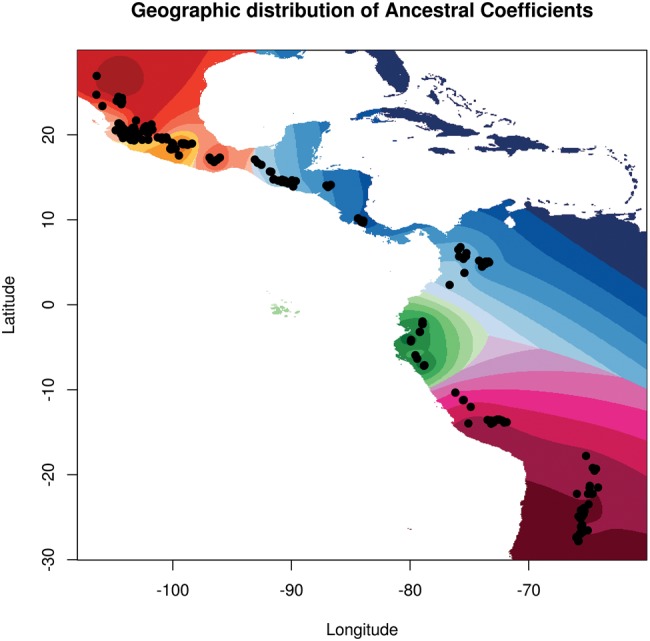
Spatial interpolation of population ancestry coefficients across the geographic distribution of the genotypes analyzed. Populations are colored as in figure 2.

Population structure analysis using the TESS3 program identified five subpopulations that best define our sample (figs. 2 and 3): three populations for the Mesoamerican (MW1 to MW3), one for the Andean (AW), and one for the Northern Peru–Ecuador (PhI) gene pool. Due to the considerable admixture in the MW group, we selected a clustering coefficient (*Q*) cut-off ≥0.7 for assigning genotypes to a specific cluster. A similar threshold was used by Bitocchi et al. (2012) while characterizing population structure in wild *P. vulgaris*.

Using this cut-off, the majority of the individuals were clearly assigned to one specific cluster, even though some genotypes where admixed with subpopulations of either the same or another gene pool (supplementary table S1, Supplementary Material online). Interestingly, the majority (∼90%) of the genotypes that could not be assigned to a specific cluster belonged to the Mesoamerican gene pool and most were admixed among MW subpopulations, especially between the two Mexican clusters MW1 and MW2. In contrast, some of them, especially wild types from Colombia, showed admixture with the Andean population (supplementary table S1, Supplementary Material online). Among the admixed individuals, there were also two of the Northern Peru–Ecuador gene pool that showed admixture with the Andean population and some Andean genotypes admixed with MW populations (fig. 2 and supplementary table S1, Supplementary Material online).

Some genotypes showed to be miss-assigned, with accession G23523 from Mexico clustering with the Northern Peru–Ecuador gene pool, the MW G23508 clustering with the Andean gene pool, and the Northern Peru–Ecuador G23584 clustering with the Andean subpopulation. Clusters MW1 and MW2 showed to be composed only of accessions from Mexico, whereas cluster MW3 was composed of accessions from Guatemala, Honduras, Costa Rica, and Colombia (fig. 3).

**Fig. 4.**
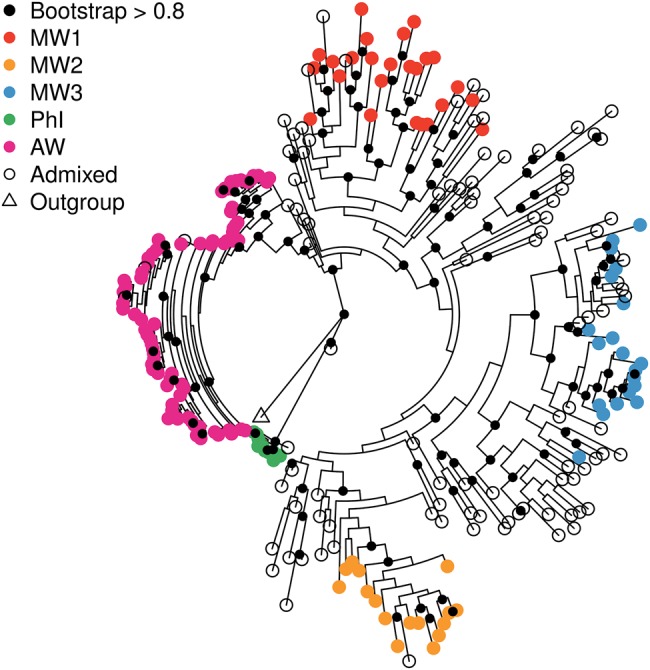
Phylogenetic tree of the wild *Phaseolus vulgaris* analyzed in the current study built using neutral variants. Populations are colored as in figure 2. Genotypes with a membership coefficient (*Q*) < 0.7, by population, analysis, were considered as admixed. *Phaseolus coccineus* PI430191 was used as outgroup for rooting the phylogenetic trees.

A similar population structure was observed in the phylogenetic tree for both the SNPs identified in genic sequences and nongenic (neutral) variants. Despite small differences beween the two phylogenetic trees, the clustering of the major gene pools of wild *P. vulgaris* showed similar patterns by using either neutral or coding variants (fig. 4 and supplementary fig. S2, Supplementary Material online, respectively). By rooting the trees with *P. coccineus* PI430191 as outgroup, the branch of the Northern Peru–Ecuador gene pool was the nearest to the tree root in both phylogenetic trees and was supported by a high bootstrapping value (>80). In addition, in both trees the nodes separating the Northern Peru–Ecuador and the AW/MW gene pools were both supported with a high bootstrap (>80), whereas the split between AW and MW populations was highly supported only in the phylogenetic tree built with neutral variants (fig. 4).

### Bioclimatic Distribution

**Fig. 5.**
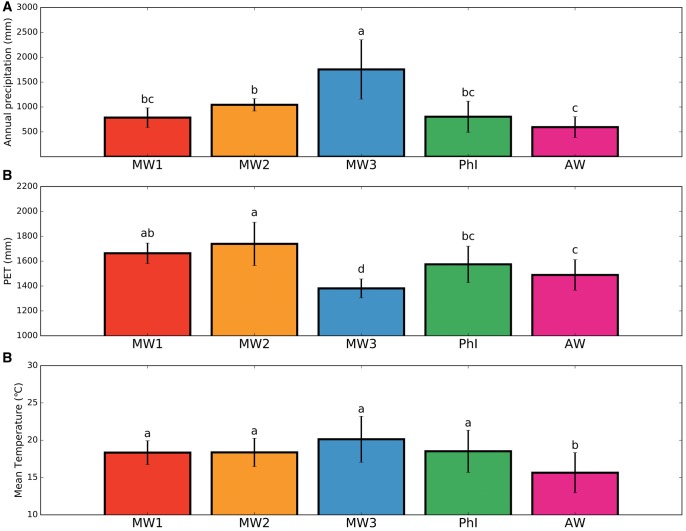
Climatic distribution of the different genetic groups identified by population clustering analysis. (*A*) Annual precipitation, (*B*) PET, and (*C*) Annual mean temperature. Only genotypes with a membership coefficient (*Q*) ≥ 0.7 were considered. The genetic groups are colored as in figure 2. Histograms represent the group mean, whereas error bars are the group standard deviation. Different letters were assigned based on Tukey–HSD multiple comparison *post hoc* test. MW, Mesoamerican wild; AW, Andean Wild; PhI, Northern Peru–Ecuador.

In order to understand if the differentiation into genetic groups of wild common bean is associated with novel environmental conditions compared with those prevalent in Mesoamerica, we characterized the climatic distribution of wild *P. vulgaris* across the genetic groups identified by TESS3. We hypothesized initially that the different genetic populations identified were subjected to similar climatic conditions and, thus, were found in similar environments. Instead, an ANOVA analysis of climatic distribution across genetic populations showed significant differences across these populations (fig. 5). For annual rainfall, the Mesoamerican genotypes of the MW3 population (located in Central America and Colombia) showed the highest value (*P *< 0.05), whereas there were no significant differences between the MW1 and MW2 populations located in Mexico. The Andean group showed the lowest annual rainfall, even though it was not significantly different than those of the North Peru–Ecuador group (PhI) and the MW1 population located in North Mexico (fig. 5*A*).

Annual potential evapotranspiration (PET) showed the highest value in the MW2 group, although no significant differences were observed between the MW1 and MW2 subpopulations (fig. 5*B*). The MW3 group showed the lowest PET, significantly different from all the other groups, whereas no significant differences were detected between PhI and AW. Annual mean temperature showed the lowest significant value in the AW group, whereas no significant differences were observed among the MW1-3 and PhI groups (fig. 5*C*).

### Demographic Modeling and Approximate Bayesian Computation

**Fig. 6.**
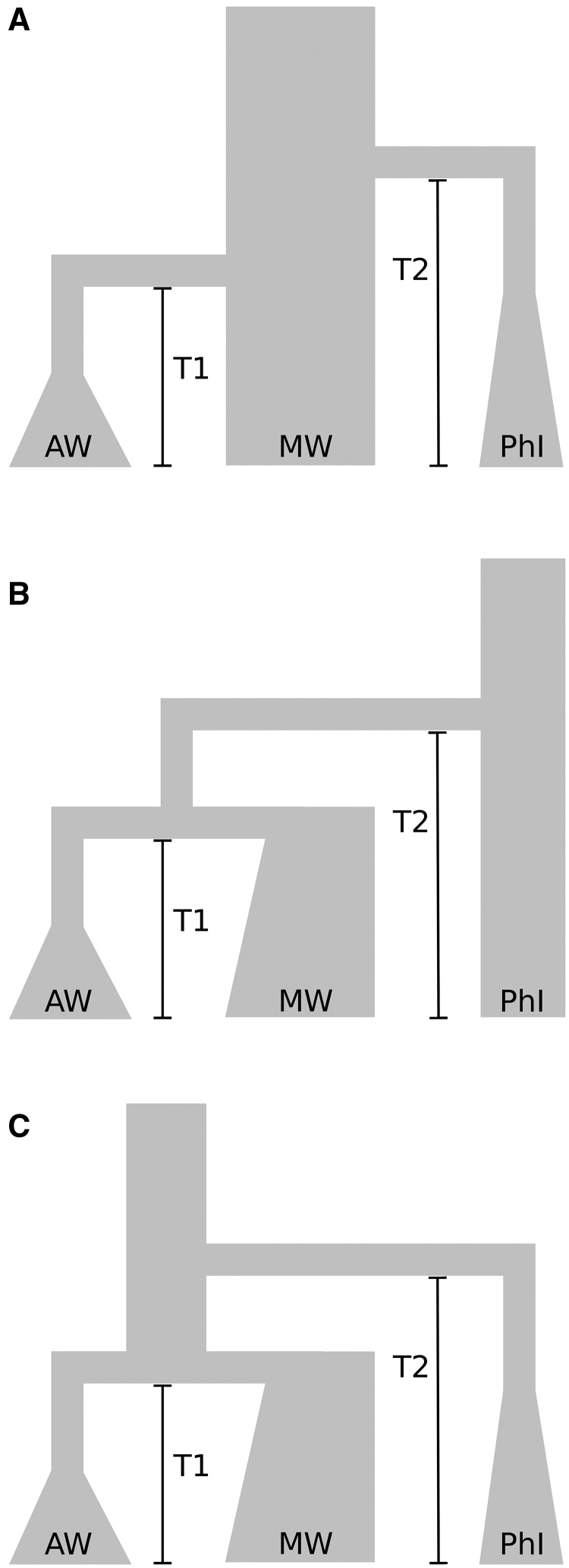
Graphical representation of the different demographic models compared in the current study. (*A*) Mesoamerican model where the Mesoamerican wild (MW) population did not experience any population bottleneck; (*B*) the Northern Peru–Ecuador model where the Northern Peru–Ecuador (PhI) gene pool did not experience any population bottleneck; and (*C*) the Protovulgaris model where the ancestral population went extinct after the Mesoamerican and Andean differentiation.

Due to the absence of a clear common ancestor between the different *P. vulgaris* wild gene pools in the collection analyzed, as observed by phylogenetic analysis, we used a constant effective population size (i.e., no signature of a bottleneck effect) as a proxy for identifying the ancestral population in our demographic modeling process (fig. 6*A* and *B*). In both of these models, the derived populations were set to undergo a bottleneck when they separated from the ancestral population. We named these two models the Mesoamerican (i.e., the Mesoamerican gene pool as ancestral population) and the Northern Peru–Ecuador (i.e., the Northern Peru–Ecuador gene pool as ancestral population) hypothesis. We also included a third model in our simulations where the ancestral population of common bean went extinct when the Mesoamerican and Andean gene pools differentiated (fig. 6*C*). We named this model the Protovulgaris hypothesis. Model comparison with approximate Bayesian computation (ABC) and population modeling was applied using 1,010,101 simulations of the different models.

Posterior probability values (PP) clearly supported the Mesoamerican hypothesis of origin of this species, with a PP of 0.96 for the standard rejection algorithm, 1 for the mnlogistic, and 0.96 for the neural network approach (table 2). Model comparison with Bayes Factor (BF) strongly supported the Mesoamerican origin of common bean for all of the approaches used, with a BF always higher than 30 when comparing the Mesoamerican with the Northern Peru–Ecuador or Protovulgaris hypothesis (table 2). Due to the higher support of the Mesoamerican hypothesis, we focused on this model for parameter inferences.

**Table 2.** Comparison between Different Demographic Models of *Phaseolus vulgaris* Evolution.

Algorithm^a^	Meso PP	Bayes Factor (Meso/PhI)	Bayes Factor (Meso/Proto)
Rejection	0.9556	63.6462	32.5382
Mnlogistic	1	1.68e^238^	4.27e^111^
NeuralNet	0.9633	121.0592	33.5401

Note.—Posterior probabilities (PP) and Bayes factors of the best model are shown. Model Name: Meso, Mesoamerican hypothesis; PhI, Northern Peru–Ecuador hypothesis; Proto, Protovulgaris hypothesis.

a Names of the algorithm used by the “abc” R package for model comparison.

Analysis of model accuracy using cross-validation showed high predictive error (>20%) for the founder population size of the Northern Peru–Ecuador and Andean groups, whereas the times of divergence showed a lower predictive error for the Northern Peru–Ecuador (<15%) and Andean (<10%) gene pools divergence time when using the 0.5% and 1% sampled points nearest to the observed summary statistic (supplementary table S2, Supplementary Material online). Due to these results, we focused mainly—for parameter inference—on the divergence times between wild common bean gene pools using the 1% sampled points nearest to the observed summary statistic. Due to the annual nature of common beans, we approximated one generation of the coalescent simulations to one calendar year for the inference of these parameters. Inferred divergence time between Andean and Mesoamerican gene pools showed an average of T1 ∼87,000 years with a 95% confidence interval (CI) in the range of 86,635–88,186 years and a highest posterior density (HPD) of the estimated divergent time between 50,008 and 168,809 years (table 3). The estimated divergence time between the Ancestral and the main common bean group showed an average of T2 ∼373,000 years (95% CI: 371,799–374,321; 95% HPD: 300,009–505,122) (table 3).

**Table 3.** Estimates of Divergence Time in Years between the Different Wild Gene Pools of *Phaseolus vulgaris* Based on ABC Analysis.

	Average (years)	95% CI^a^ (years)	95% HPD^b^ (years)
PhI divergence time	373,060	371,799, 374,321	300,009, 505,122
AW divergence time	87,410	86,635, 88,186	50,008, 168,809

Note.—PhI, Northern Peru–Ecuador gene pool; AW, Andean gene pool.

a 95% confidence interval around the mean.

b 95% highest posterior density of the sampled distribution.

## Discussion

The distribution of what is currently known as wild *P. vulgaris* is nothing short of extraordinary, especially among crop plants. Indeed, this distribution extends from northern Mexico [G23463 (GN 84154): Yepachic, Chihuahua, Mexico; collected by G.P. Nabhan; 28.3 N. Lat., −108.5 W. Long.] to northwestern Argentina (Punilla, Córdoba, Argentina; collected by Sirolli et al. 2015; −31.3 S. Lat., −64.6 W. Long.). Information about this distribution has been gained during explorations that have been conducted since the 1940s. From initial observations in Guatemala, Honduras, and Argentina (McBryde 1947; Burkart and Brücher 1953; Berglund-Brücher and Brücher 1976; Brücher 1988), further systematic explorations, among others by Gentry (1969), Miranda Colín (1967), Debouck (1991), Toro et al. (1990), Freyre et al. (1996), Freytag and Debouck (2002), and Sirolli et al. (2015), have provided additional information on the overall distribution of wild common bean.

This distribution is not continuous but shows some significant gaps (Chacón et al. 2007; Rodriguez et al. 2016). Aside from short-distance topographic gaps (such as deep valleys or high volcanic slopes), such as around the Colima (Mexico) and Chimborazo (Ecuador) volcanoes, wider gaps are generally found in lower or higher altitude areas or traverse the Andes mountain range. These gaps include the Isthmus of Tehuantepec in Mexico, the country of Nicaragua, the Isthmus of Panama, and the adjacent Chocó region of Colombia. In southern Colombia–northern Ecuador, there is a gap where the distribution of these wild populations switches from the eastern slopes of the Andes (to the North) to the western slopes of this range (to the South). An additional gap is located in central Peru where the distribution switches back from the western side to the eastern side of the Andes and between southern Peru and northern Bolivia (Lake Titicaca region). In general, the main variables determining these gaps are climatic variables related to rainfall and temperature.

In this research, we obtained additional information on the genotypic differentiation of the wild common bean populations and their environmental distribution. We developed a broad sample of wild *P. vulgaris* accessions, representing most of the extensive geographic distribution of this wild progenitor and used the GBS genotyping approach, which resulted in some 20,000 SNPs. This number of markers is much larger than what has been used before in this type of diversity study, especially as no imputation was used. Whereas imputation is a valid probabilistic approach in segregating populations when parental genotypes are known, imputation may lead to experimental overreach, in that information on the origin of a nucleotide identity (whether by sequencing or imputation) may get lost. In addition, genotype imputation in data sets with a relatively high percentage of missing data, such as GBS, increases the number of false heterozygous calls (Xavier et al. 2016), making it unreliable for our study and for species like common bean that show a high level of homozygosity even in the wild (Kwak and Gepts 2009; Blair et al. 2012). Therefore, we prefer not to conduct imputation in genetic diversity studies such as this one.

Our results confirm the subdivision of the wild *P. vulgaris* gene pool into three major subgene pools (Koenig and Gepts 1989; Debouck et al. 1993; Freyre et al. 1996; Kwak and Gepts 2009). There were no differences in the two Andean gene pools between Kwak and Gepts (2009) and the current study: K1 and K7 of Kwak and Gepts (2009) corresponded to the PhI and AW gene pools, respectively, of the current study. Although Kwak and Gepts (2009) also identified further subdivisions in the Mesoamerican wild gene pool, there were differences in the geographic distributions of their two subgroups and the ones identified in this study. Kwak and Gepts (2009) identified a purely Mexican wild group (their K5) composed of accessions from central and northern Mexico and a second group, which included entries from central Mexico, Guatemala, and Colombia (their K3). Differences with the current study are likely due to changes in plant sampling, especially a more extensive sampling in the Mesoamerican gene pool in the current study. In addition, these differences could also be caused by the improvement of genotyping technology, which increased the number of markers analyzed, along with the development of novel population clustering algorithm optimized for high-throughput genotyping data.

The three major gene pools showed different values for key population genetic variables. The Mesoamerican gene pool was the most diverse with a *π* value of = 1.42e^−5^, compared with *π *= 1.05e^−5^ and 6.46e^−6^ for the PhI and Andean gene pools, respectively (table 1). The reduced diversity observed in the PhI and AW gene pools compared with Mesoamerican gene pools is similar to that obtained by other authors (Bitocchi et al. 2012; Mamidi et al. 2013; Schmutz et al. 2014) but cannot be compared precisely with other studies because of different gene pool concepts or sample coverages for accessions or sequences. For example, Schmutz et al. (2014; fig. 2) did not include wild accessions between Honduras and northern Peru in their diversity calculations. Bitocchi et al. (2012) also observed that the PhI group had higher diversity than the AW group (*π* = 2.7e^−3^ vs. 1.0e^−3^, respectively). Nevertheless, the lower diversity levels in the PhI and AW groups are due in part to the very narrow habitat they occupy on the western and eastern flanks, respectively, of the Andes Mountains. The other cause is, of course, the long-distance dispersal leading to genetic drift and selection.

A similar pattern was observed in the number and size of haplotype blocks in the three wild gene pools, with the Mesoamerican having the shortest haplotype block lengths, and the Andean the longest one. Studies of haplotype blocks in human populations showed shorter haplotype blocks length in African population when compared with non-African ones, suggesting that population demography and evolution could shape the pattern of haplotype blocks length across the genome (Gabriel et al. 2002). These results suggest that a similar phenomenon obviously occurs in plants genomes as well. These variations observed between the three gene pools can be attributed to several nonmutually exclusive causes, including a larger effective population size in Mesoamerica, different ages since migration from the Mesoamerica into the Andes, and founder effects due to these migrations.

Correlations between annual means of climate variables and the distribution of the five population groups (MW1, MW2, MW3, PhI, and AW; fig. 5) showed that the MW3 group (Southern Mexico, Central America, and Colombia) is subjected to the highest level of rainfall compared with the other groups, whereas the Andean group AW received the least precipitation. With regard to temperature, the three Mesoamerican wild groups and the PhI group grow in similar average temperatures, which were higher than those for the AW group. PET was the highest for the MW1 and MW2 groups, followed by the two Andean groups (PhI and AW), and, not surprisingly, least for the MW3 group. These significant variations of important climatic variables observed between the different genetic populations identified in wild common bean suggest that these subpopulations are probably the result of adaptation to new environments, instead of random drift. The habitat of wild *P. vulgaris* is located within the seasonally dry, Neotropical forest, one of the most threatened biomes on Earth (Banda-R et al. 2016). Thus, one might have expected similar environments in this habitat. Nevertheless, Banda-R et al. (2016) also documented 12 floristic groups within this dry forest, suggestive of different environments. In particular, groups MW1 and MW2 are found in the Mexican floristic province, MW3 in the Central America–Northern South America and Northern inter-Andean Valleys provinces, PhI in the Central inter-Andean Valleys province, and AW in the Piedmont and Apurimac-Mantaro provinces.

More specifically, variations in water-related traits, such as drought tolerance or water-use efficiency, may be more common in the MW1 and MW2 groups, and the derived domesticates, as observed in the eco-geographic race Durango (Singh et al. 1991). In contrast, these variations were also consistent with the adaptation to relatively cooler and moister environments encountered by Andean wild beans and their domesticates, for example, in Colombia (Debouck et al. 1993) and, following dispersal out of the southern Andean center of domestication, in western Europe in general (Gepts and Bliss 1985) and, for example, the Netherlands (Zeven 1997). Variation in these climatic variables among genetic groups also provides a rough estimate of the increase in ecological amplitude and the potential for adaptation to changing climatic conditions, a species such as *P. vulgaris* was and will be exposed to. Over the entire range, wild *P. vulgaris* is exposed to a 3-fold range in average rainfall (∼500–1,500 mm), a range of ∼4 °C (an increase of ∼2 °C for the MW3 dispersal and a decrease of ∼2 °C for the AW dispersal). The three long-distance dispersal all displayed a decrease in PET (∼300, 100, and 200 mm, respectively). Any need for adaptation beyond these ranges will probably require genetic diversity contained in other *Phaseolus* species, as illustrated by the adaptation to higher temperature provided by *P. acutifolius* and *P. lunatus* (Medina et al. 2017).

Because the genus *Phaseolus* originated some 5–6 Ma in what is now called Mesoamerica (Delgado-Salinas et al. 1999), it follows that the MW, PhI, and AW gene pools also ultimately originated there following dissemination from this core area (Chacón et al. 2007). However, it was not clear until the current research how the contemporary distribution was achieved: for example, how many dissemination events, whether single or repeated, took place and when. The phylogenetic analysis conducted on the SNP diversity identified in this study, showed no nesting of the PhI group inside the combined wild Andean and wild Mesoamerican clade (A + M groups), or even within the Mesoamerican clade (fig. 4). In both of the phylogenetic trees, based on genic and nongenic variants (supplementary fig. S2, Supplementary Material online and fig. 4, respectively), the PhI group behaved mostly as an intermediate taxon between *P. coccineus* (used as outgroup) and the A + M clade.

Further evidence for the distinctness and older age of the PhI group compared with the A + M groups is provided by Rendón-Anaya, Montero-Vargas, et al. (2017), who also placed PhI individuals (called Amotape-Huancabamba group by them) in a separate clade that is sister to the A + M group based on both nuclear SNPs, using a next-generation-sequencing-based approach, and chloroplast DNA (cpDNA) sequences. The divergence time of the PhI (AH) group with the A + M groups was estimated at 0.26 and 0.9 My based on nuclear and cpDNA data, respectively, well before their estimate for the separation between the AW and MW gene pools (0.002 and 0.2 My based on nuclear and cpDNA data, respectively). Furthermore, metabolomic analyses separated the PhI group from the A + M group as well and placed the PhI group closer to the *P. coccineus* species. Based on these results, Rendón-Anaya, Montero-Vargas, et al. (2017) proposed that the PhI group was actually a sister or cryptic species of *P. vulgaris* and named this species *Phaseolus debouckii* (Rendón-Anaya, Herrera-Estrella, et al. 2017). Chacón et al. (2007) observed that the PhI group included two unique cpDNA haplotypes, which were absent in all the accessions of either the Mesoamerican and Andean wild gene pools of *P. vulgaris.*Kami et al. (1995) observed that the phaseolin genes, coding for the main seed storage protein, of the PhI group were ancestral to those observed in *P. vulgaris*, because of the absence of tandem direct repeats in the PhI group and their presence in the latter. Such absence was also observed in *P. coccineus* and *P. dumosus*, the most closely related species belonging to the *vulgaris* group (Delgado-Salinas et al. 1999). In contrast with Kami et al. (1995), who posited that the PhI group was a direct ancestor of the A and M gene pools, we now propose—based on all available evidence—that the PhI group shares a common ancestor with the A + M group. This common ancestor remains to be discovered or has become extinct. If still extant, it may have a morphology quite similar to Mesoamerican wild *P. vulgaris*, but carry an I-type phaseolin.

A similar observation could be made for the divergence between the southern Andean and Mesoamerican wild beans, which pointed to the absence of a common ancestor between the MW1, MW2, and MW3 groups, in one clade, and the AW group in the other clade. Indeed, the majority of previous studies performed for understanding common bean evolution clearly showed that the Andean gene pool evolved from the Mesoamerican group. However, all those studies focused on a reduced set of molecular markers (or on subset of SNPs located within gene sequences) instead of using a set of SNPs widely distributed across the genome as in the current study. The lack of a common ancestor in the phylogenetic tree between MW and AW, along with the strong *F*_st_ value between these two groups, suggests that the actual ancestor of wild *P. vulgaris*, presumably located in Mesoamerica, could have gone extinct. It also supports the hypothesis that the Mesoamerican and Andean pools of this species are undergoing an incipient speciation event due to geographic isolation among these groups. Indeed, hybrid weakness of F1 individuals has been observed in crosses between Mesoamerican and Andean genotypes, belonging either to the wild and domesticated gene pools of this species (Gepts and Bliss 1985; Koinange and Gepts 1992). This interpool incompatibility predates domestication, and is determined by two complementary genes that evolved separately in the MW and AW gene pool (Shii et al. 1980; Gepts and Bliss 1985; Hannah et al. 2007).

Although our ABC analysis supported a Mesoamerican origin of common bean (confirming the findings of Bitocchi et al. 2012), it also estimated that the PhI group separated from the main common bean group some ∼0.37 Ma (between 0.3 and 0.5 Ma at 95% Highest Posterior Density), >0.28 My before the divergence of the MW and AW group (∼0.087 My, 0.05–0.168 HPD 95%). These estimates are earlier than previously observed by analyzing internal transcribed spacers of ribosomal DNA on a subset of ten common bean from different gene pools, but fall within the observed divergence interval (Chacón et al. 2007). Nevertheless, these estimates are concordant with those observed with nuclear markers while comparing 12 species of the *Phaseolus* genus (Rendón-Anaya, Montero-Vargas, et al. 2017).

Given that the core area of the entire genus *Phaseolus* as defined by Maréchal et al. (1978) is located in Mesoamerica (Delgado-Salinas et al. 1999; Freytag and Debouck 2002), the existence of species or populations outside this area suggests one or more long‐distance dispersal (LDD) events. The existence of the genetic subgroups within the collection, and the admixture within these subgroups, provided an initial clue as to the number and range of these LDD events. In general, we propose that the current geographical distribution of wild *P. vulgaris* has been achieved by seed dispersal at three spatial scales, each of them associated with its own temporal scale. At close range (within a few meters), bean plants disperse their seeds through the explosive dehiscence of their pods. The dehiscence phenomenon lies at the intersection of genetic (Suzuki et al. 2009; Lenser and Theißen 2013; Balanzà et al. 2016), anatomic pod structure (Prakken 1934), and environment factors that promote seed dispersal in wild beans but were selected against during domestication (Koinange et al. 1996). This dispersal takes place in each of the gene pools and each year, and could increase the possibility of gene flow between sympatric populations growing in the same areas. A similar phenomenon has been observed between wild and domesticated common bean of the Mesoamerican gene pool (Papa and Gepts 2003) or within domesticated maize and the wild *Zea mays* subspecies *mexicana* (Heerwaarden et al. 2011). The high admixture observed between individuals of the two Mexican subpopulations (MW1 and MW2), could be the direct result of this short-range dispersal.

At medium range (from a few meters to hundreds of km), the seeds are dispersed over a contiguous landscape composed of adjacent or sympatric suitable habitats. Although dispersal may not happen every year, it can take place relatively frequently allowing a certain degree of admixture between relatively distant populations and limit differentiation and, eventually, speciation. Potential agents of seed dispersal may be rodents, birds, and megafauna (Sandom et al. 2014; Pires et al. 2017). This medium-range dispersal could be responsible for the current distribution of each of the STRUCTURE groups (MW1-3, PhI, and AW), as well as the admixture observed within the Mesoamerican gene pool, especially between the MW3 and MW1-2 groups, or the admixture between MW3 genotypes located in Colombia and AW groups.

LDD bridges gaps imposed by unsuitable environments. In the case of wild common bean, these are either lowlands, consisting mainly of humid and hot areas, or highlands (see earlier). Our data suggest then that the current distribution of wild *P. vulgaris* is the result of at least three long-distance migrations (PhI, AW, and MW3, in decreasing order of divergence and age) from a core ancestral area in Mesoamerica. Other *Phaseolus* species have been subjected to these seed dispersal at different spatial scales. These include, for example, lima bean (*P. lunatus*), which has a wild progenitor distribution similar in magnitude to that of wild common bean, as it extends from Mexico to Argentina and was also domesticated twice (Salgado et al. 1995; Fofana et al. 2001; Serrano-Serrano et al. 2010). They also include, for example, the nondomesticated species *P. polystachyus*, distributed in the eastern USA and the Caribbean, and *P. mollis* on the Galapagos Islands.

This type of LDD across different type of habitats suggests a role for migrating birds. Some wild bean populations in Ecuador are known as *frijol de paloma* (dove or pigeon bean) as birds damage pods to extract seeds from them (Debouck et al. 1993). In recent years, the migration of birds and their role as LDD agents has been highlighted (Remsen 1984; Sorte et al. 2016; Viana et al. 2016). Further data are needed to corroborate this hypothesis; however, it is clear that such long-distance, habitat-gap-bridging migration events are rare. Our data suggest that these events only occur on a time scale of the order of 100,000 years. Thus, distance and frequency of seed dispersal are inversely correlated.

On a broader level, LDD events are keys element influencing plant population structure, evolution, diversity, and finally also the ability to colonize new habitats (Cain et al. 2000; Bialozyt et al. 2006). Due to the difficulties in measuring long-range dispersal events empirically, several simulations model has been developed for estimating how these events influence plant diversity and population structure (Cain et al. 2000; Nathan et al. 2003; Bialozyt et al. 2006). A study from Bialozyt et al. (2006) identified close relationships between the frequency of LDD events, the size of the colonized areas, and the effect on genetic diversity. In particular, when these events are very rare and the colonized region is narrow, the resulting population showed a complete loss of genetic diversity and increase in genetic uniformity, similar to what we observed for AW and, to a lesser extent, PhI. The authors called this phenomenon the “embolism effect.” On the other hand, when these events are more frequent and the colonized areas are wider, the genetic diversity of the resulting population is maintained. This phenomenon is named “reshuffling effect.” Even though the “embolism” and “reshuffling effect” were first hypothesized using computer simulation modeling, these concepts help explain patterns previously observed in wild plant populations (Liepelt et al. 2002; Petit et al. 2002, 2004; Stenøien et al. 2005).

Our data further confirm these hypotheses, but also highlight gene-pool-specific LDD patterns in wild *P. vulgaris*. The PhI and AW were subjected to the “embolism effect” as the result of a single LDD event toward narrow habitats with modest to little range expansion after the initial colonization, consistent with the eco-geography and topography of the Andes mountains. On the other hand, the MW3 population structure and diversity could be the result of the “reshuffling effect,” where multiple LDD events and continuous range expansions maintained genetic diversity outside the center of origin.

The frequency of LDD events of PhI, AW, and MW3 groups could have been driven by bird migration patterns across the Americas. Indeed, a recent survey of broad-scale migration strategies of terrestrial birds in the Western Hemisphere showed that the majority of migration trajectories were concentrated in the Northern Hemisphere of the Americas (Sorte et al. 2016).

## Conclusion

In the present study, the genotyping at a genome-wide level of some 250 individuals representative of wild common bean populations resulted in a more complete and comprehensive characterization of the genetic diversity, relatedness, and evolution of this species in comparison with previous studies. In addition, the integration of genetic and ecological data enabled the identification of patterns of dispersal and colonization leading to the extensive distribution of the wild ancestor of this crop across the Americas. It also clarifies that these migration has led to colonization of regions with different climates than that of the region of origin (based on annual temperature, rainfall, and PET). Furthermore, this distribution carries the unusual distinction that it includes the descendant of a potentially extinct ancestor (the PhI group of Ecuador and northern Peru) outside the ancestral area of the genus *Phaseolus* in Mesoamerica. An improved characterization of the phylogeny and ecological distribution of wild common‐bean leads to a better understanding of the genetic diversity and adaptation of the two domestications in this species (Mesoamerica and southern Andes).

## Materials and Methods

### Plant Materials

A panel of 271 wild *P. vulgaris* accessions covering the entire geographical distribution of common bean from Northern Mexico to Northwestern Argentina were selected. All three gene pools of common bean were represented in our panel, with 175 accession of the Mesoamerican (MW), 84 of the Andean (AW), and 12 of the ancestral (PhI) gene pool from Northern Peru and Ecuador, the latter group characterized by the ancestral type I phaseolin (Kami et al. 1995). In addition, other wild accessions of five *Phaseolus* species were analyzed including four *P. lunatus*, two *P. dumosus*, two *P. acutifolius*, two *P. coccineus*, and two *P. augusti*, and two domesticated common bean accessions as internal control. The list of the accessions sequenced, together with their respective gene pools, species information, and georeferenced coordinates, is available in supplementary table S3, Supplementary Material online. Seeds of these accessions were provided by the International Center of Tropical Agriculture (CIAT, Cali, Colombia) and the United States Department of Agriculture Western Regional Plant Introduction Station (Pullman, WA).

### Library Preparation and Illumina Sequencing

DNA extraction and GBS Illumina library preparation followed the GBS protocol developed for common bean and described in Ariani et al. (2016). Prior to library preparation, DNA quality was checked with NanoDrop Lite (Thermo Fisher Scientific) and by 1% agarose gel electrophoresis. DNA with an absorbance ratio (A260/A280) > 1.7 and with no visible degradation on agarose gel was used for subsequent library preparation. Genomic DNA was quantified with Quant-iT PicoGreen dsDNA Assay Kit (Thermo Fisher Scientific) and 10 ng of DNA was used for NGS library preparation. The presence of adapter dimers in the sequencing libraries was checked with Experion DNA analysis kit (Biorad, Berkely, CA). Samples were multiplexed in two libraries with 144 genotypes in each library. As internal control, a blank sample and the *P. vulgaris* genotype used to determine the reference genome sequence (G19833) were included in each of the two libraries. Specific barcodes and adapters for *Cvi*AII were designed with the GBS barcoded adapter generator (http://www.deenabio.com/services/gbs-adapters; last accessed October 26, 2017). The list of the barcode sequences used for multiplexing, the relative genotypes and the SRA accession number for each library are shown in supplementary table S3, Supplementary Material online.

The two libraries were first evaluated in one Illumina HiSeq2500 lane each to check the effectiveness of multiplexing at the UC Davis Genome Center, and then sequenced in four other Illumina HiSeq2500 lanes each at the QB3 Vincent J. Coates Genomics Sequencing Laboratory at the University of California, Berkeley, CA. All the sequencing was performed with the 100-bp single-end Illumina protocol. Raw reads are available at the NCBI Sequence Read Archive (http://www.ncbi.nlm.nih.gov/sra; last accessed October 26, 2017) under the accession numbers SRX2771627 and SRX2771628.

### Bioinformatic Data Analysis

Reads were preprocessed by clipping the reads containing the full restriction site (CATG) or the adapter contaminants, and by quality trimming using a 5-bp sliding window and a cut-off quality (averaged on the 5-bp window) of 20. Reads longer than 30 bp after the preprocessing, and containing the overhang sequence of *Cvi*AII digestion (i.e., ATG) after the barcode sequence, were then demultiplexed allowing no mismatch on the barcode sequence. Demultiplexed reads were aligned to the *P. vulgaris* reference genome sequence (accession G19833; Schmutz et al. 2014) using BWA mem algorithm (Li and Durbin 2009) and only reads with a minimum mapping quality of ten were used for variant calling. Variants were called using SAMtools (Li et al. 2009) and final filtering was performed with VCFtools (Danecek et al. 2011). After SNPs calling, only genotype calls with a minimum quality of 10 (-minGQ 10), a minimum read depth higher than three reads (-minDP 3), and located outside repetitive regions were retained.

For analysis of genetic diversity in wild *P. vulgaris*, all the other *Phaseolus* species and the domesticated genotypes were removed from the data set prior to SNP filtering. In the last filtering step, we removed the genotypes with <10% of genotyped positions (-mind 0.1), kept only biallelic SNPs with <20% of missing data (-geno 0.8) and with a Minor Allele Frequency (MAF) higher than 0.05 (-maf 0.05). Variant statistics were calculated using VCFtools. Nucleotide diversity, Tajima’s *D* (Tajima 1989), and *F*_st_ (Hudson et al. 1992) were calculated and averaged on 100-kb genomic bins containing at least three variants. Haplotype blocks were identified with PLINK (v1.07) (Purcell et al. 2007), for the entire sample and for the separate gene pools, using SNPs no more than 2 Mb apart (–ld-window-kb 20000). To avoid the bias introduced by nearby SNPs, only blocks longer than 100 bp were taken into account for comparisons between gene pools. The haplotype blocks across the entire sample and within the single gene pools where compared by evaluating the total number of blocks and their N50.

### Molecular PCA, Genetic Structure, and Phylogenetic Analysis

Molecular PCA was performed using the adegenet package (Jombart 2008) implemented in the R environment. Spatial population structure was inferred with TESS3 (Caye et al. 2016), an algorithm specifically developed for inferring population structure using both genetic and geographic data. The program was run with the number of populations (*K*) ranging from 2 to 10 and 20% of masked genotypes (for computing the cross-entropy criterion). After manual evaluation of population clustering and geographic distribution of these populations for each value of *K*, we selected *K* = 5 as the number of population best representing the collection analyzed.

Phylogenetic inference was performed with SNPhylo (Lee et al. 2014), analyzing separately the variants located in genic sequences and those at least 5 kb from an annotated feature in the *P. vulgaris* genome (presumably neutral variants). SNPhylo has been specifically designed for reconstructing phylogeny from SNPs data; it is able to collect representative SNPs for each linkage block, based on LD, in order to reduce marker redundancy. With both of these data sets, we selected an LD threshold of 0.4 for reducing marker redundancy in SNPs analysis. For both data sets, we performed alignments with the MUSCLE algorithm (Edgar 2004) and inferred the phylogenetic tree using Maximum Likelihood with 1,000 bootstrap replicates. As outgroup for rooting the phylogenetic tree the accession PI430191 (*P. coccineus*) was used.

### Bioclimatic Distribution

An analysis of the climatic distribution of the final 246 individuals retained for the final analysis was performed within the R statistical environment (www.r-project.org; last accessed October 26, 2017). Based on the geographical coordinates of the genotypes analyzed, we extracted climatic variable using the dismo R package. Annual rainfall and annual mean temperature were extracted from the WorldClim database (http://www.worldclim.org/; last accessed October 26, 2017) at 30 sec resolution. Annual PET was extracted from the Global Aridity and PET database (http://www.cgiar-csi.org/data/global-aridity-and-pet-database; last accessed October 26, 2017). For identifying possible variations of climatic variables within the genetic groups identified by TESS3, we performed an analysis of variance (ANOVA) using only genotypes with a membership coefficient ≥ 0.7. *Post hoc* multiple comparisons were performed with the Tukey’s HSD test implemented in the agricolae R package.

### Demographic Modeling and ABC

Due to the ongoing debate regarding the origin of common bean as a species, we performed a demographic modeling and an ABC analysis to identify the best evolutionary model of evolution for this species. For this analysis, only the neutral variants identified in this study were taken into account. These variants were converted with PGDSpider2 (Lischer and Excoffier 2012) into a diploid Arlequin file format (Excoffier and Lischer 2010) and summary statistics were computed using arlsumstat with default parameters (Excoffier and Lischer 2010). We compared three different models, namely the Mesoamerican, Northern Peru–Ecuador and Protovulgaris models (see Results). For model comparison, we generated 1,010,101 simulations with the ABCsampler program of the ABCtoolbox suite (Wegmann et al. 2010) by sampling parameters from a defined prior distribution for each model (supplementary table S4, Supplementary Material online). Coalescent simulations were performed with 1,000 SNPs on five diploid chromosome with a MAF of 0.05 using the fastsimcoal2 simulator (Excoffier et al. 2013). Summary statistics were computed with arlsumstat using default parameters. The best model was selected using the abc package available on R (Csilléry et al. 2012) by evaluating the PP and the Bayes factors between the three simulations. For comparison purpose, we used the 1% sampled point nearest to the observed summary statistic and either the standard rejection approach, the multinomial logistic regression (“mnlogistic”), and the neural networks (“neuralnet”) algorithm available in the package. With the neural networks algorithm, we used 100 neural networks and 1,000 iterations.

Demographic parameters were estimated with the abc package available on R (41). Prior to parameter inferences, we evaluated the accuracy of prediction of our model by cross-validation, implemented in the R package, on a subset of 1,000 randomly sampled simulations using the standard rejection algorithm. We evaluated this accuracy on three tolerance levels, using the 0.5%, 1%, and 5% sampled points nearest to the observed summary statistic. We inferred the time of divergence between the Ancestral and the main common bean group, and between the Andean and Mesoamerican gene pools using the standard rejection algorithm and the 1% sampled points nearest to the observed summary statistics. Confidence interval and highest posterior density (HPD) of the means of this points for each divergence time were calculated using the SciPy (http://www.scipy.org/; last accessed October 26, 2017) and DendroPy (Sukumaran and Holder 2010) python libraries, respectively.

## Supplementary Material

Supplementary data are available at *Molecular Biology and Evolution* online.

## Supplementary Material

Supplementary Data
