# Supplementary material for: Spatial and Temporal Scales of Range Expansion in Wild Phaseolus vulgaris
Source: Mol Biol Evol. 2017 Oct 23;35(1):119–31. doi: 10.1093/molbev/msx273 (PMC5850745; doi:10.1093/molbev/msx273)
Supplement: Supplementary Data [file msx273_supp.zip › Ariani et al. Suppl. files.pdf]

Ariani et al. **Spatial and Temporal Scales of Range Expansion in Wild *Phaseolus vulgaris***

Supplementary Figures

Figure legends:

Fig. S1: Distribution of genes and SNPs density across the 11 common bean chromosomes

Fig. S2: Phylogenetic trees of the wild *P. vulgaris* analyzed in the current study built using genic variants. Populations are colored as in Fig. 2. Genotypes with a membership coefficient (Q) < 0.7, by population structure analysis, were considered as admixed. P. coccineus PI430191 was used as outgroup for rooting the phylogenetic tree.

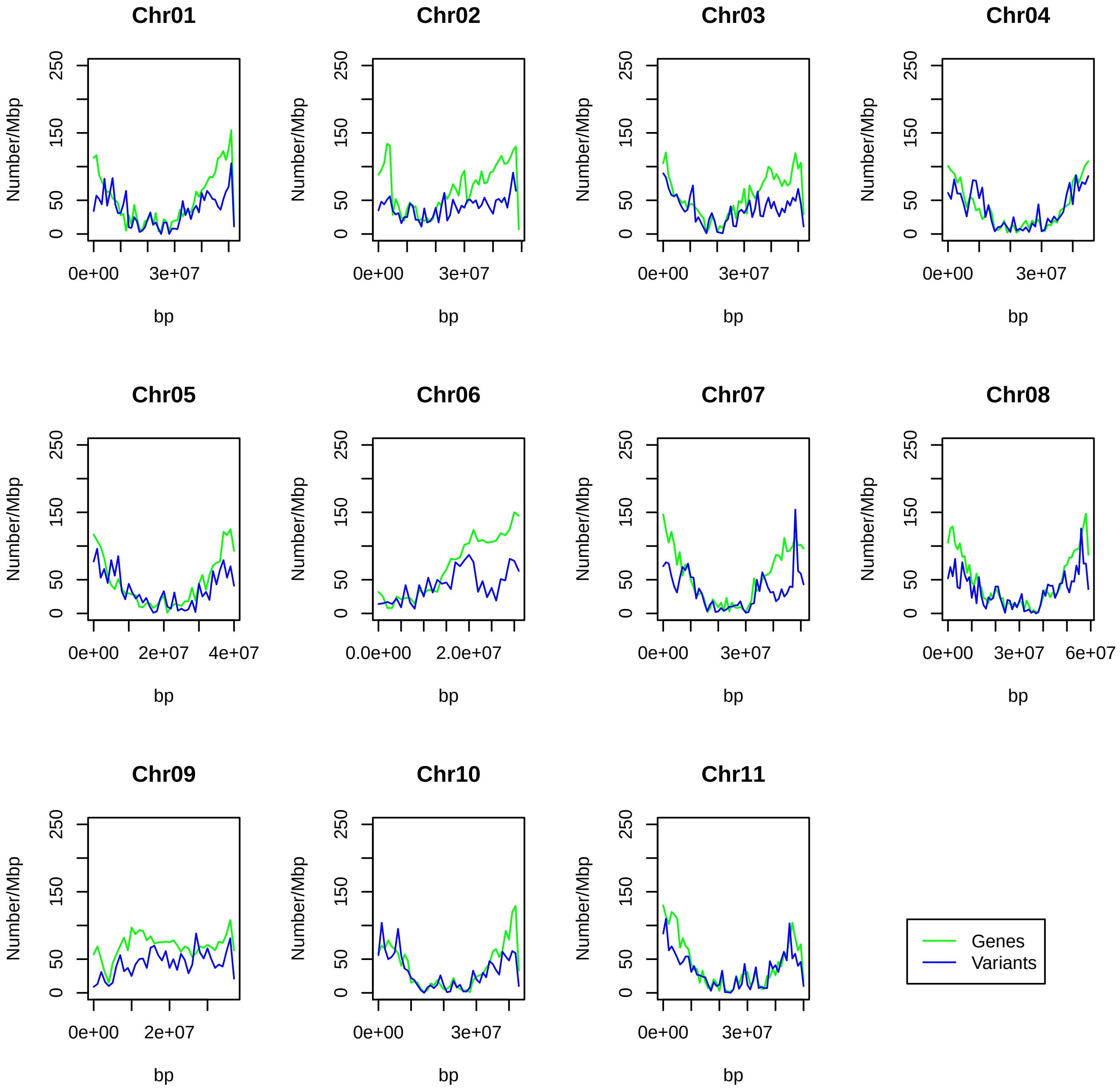
Chr01
Chr02
Chr03
Chr04
Chr05
Chr06
Chr07
Chr08
Chr09
Chr10
Chr11
Number/Mbp
bp
0
50
150
250
0e+00
3e+07
2e+07
4e+07
6e+07
0.0e+00
2.0e+07
Genes
Variants

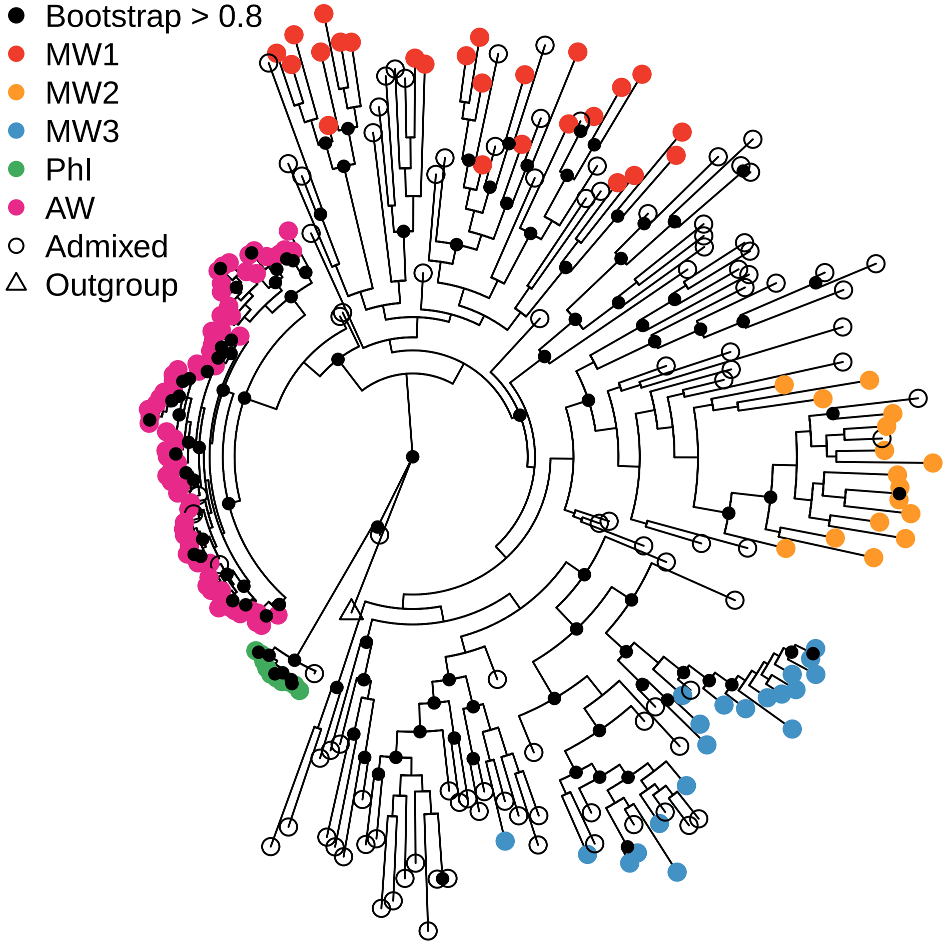
Bootstrap > 0.8
MW1
MW2
MW3
Phl
AW
Admixed
Outgroup
