# Supplementary material for: Spatial and Temporal Scales of Range Expansion in Wild Phaseolus vulgaris
Source: Mol Biol Evol. 2017 Oct 23;35(1):119–31. doi: 10.1093/molbev/msx273 (PMC5850745; doi:10.1093/molbev/msx273)
Supplement: Supplementary Data [file msx273_supp.zip › Table_S1.pdf]

Table S1: List and population clustering resultsof the final wild *Phasolus vulgaris* analyzed in this study
Accession ID, country of origin,geographical cordinates of collection, and gene pools information are shown.

Accession	Country	Lat	Long	Gene Pool[1]	Cluster [2]
E2111	Costa Rica	9.8666	-84.1166	MW	MW2
E3121	Costa Rica	10.1666	-84.3833	MW	MW2 (0.63), AW (0.16)
E3132	Costa Rica	10.1666	-84.3833	MW	MW2
E3134	Costa Rica	9.8	-84.1166	MW	MW2
E3136	Costa Rica	9.85	-84.0666	MW	MW2
E3168	Costa Rica	9.65	-83.95	MW	MW2
E3178	Costa Rica	9.85	-84.0333	MW	MW2
E3186	Costa Rica	9.7333	-84.0833	MW	MW2
G10011	Mexico	18.8833	-99.15	MW	MW3
G10018	Mexico	19.6833	-100.9167	MW	MW3
G10019A	Mexico	19.4667	-100.4833	MW	MW1 (0.43), MW3 (0.40)
G11028	Mexico	24.4667	-104.5833	MW	MW1
G11050A	Mexico	19.6833	-101.2667	MW	MW1
G11051	Mexico	20.7667	-103.4	MW	MW1
G11052	Mexico	20.8	-103.4	MW	MW1
G11053	Mexico	20.85	-103.2667	MW	MW1
G11115	Mexico	19.4	-103.4	MW	MW1 (0.42), MW3 (0.41)
G12851	Guatemala	14.2833	-90.3	MW	MW1 (0.31), MW2 (0.30), MW3 (0.27)
G12852	El Salvador	13.9167	-89.85	MW	MW2 (0.40), MW1 (0.30)
G12856	Peru	-10.3333	-76.1833	AW	AW
G12857	Peru	-11.2167	-75.4833	AW	MW1 (0.40), AW (0.29), MW3 (0.14)
G12860	Mexico	23.8833	-104.2667	MW	MW1
G12861	Mexico	19.4167	-102.0667	MW	MW3 (0.49), MW1 (0.26)
G12863	Mexico	20.7833	-104.1833	MW	MW1 (0.58), MW3 (0.32)
G12864	Mexico	19.4667	-103.5833	MW	MW1 (0.48), MW3 (0.32)
G12865	Mexico	19.3333	-103.25	MW	MW3
G12866	Mexico	19.6833	-103.4833	MW	MW1 (0.61), MW3 (0.35)
G12867	Mexico	21.0833	-104.5	MW	MW1 (0.44), MW3 (0.36)
G12868	Mexico	21.3333	-104.5833	MW	MW3 (0.48), MW1 (0.35)
G12869	Mexico	19.4167	-102.5833	MW	MW3 (0.49), MW1 (0.26)
G12870	Mexico	23.3833	-105.9333	MW	MW1 (0.40), MW3 (0.33)
G12872	Mexico	18.9667	-99.1	MW	MW3
G12873	Mexico	19	-99.25	MW	MW3
G12875	Mexico	17.3167	-96.9	MW	MW1
G12877	Mexico	18.95	-99.2167	MW	MW1 (0.64), MW 3 (0.18)
G12878	Mexico	18.35	-99.7667	MW	MW3 (0.46), MW1 (0.29)
G12879	Mexico	18.35	-99.9833	MW	MW3 (0.52), MW1 (0.15), MW3 (0.15)
G12882B	Mexico	18.2833	-100.15	MW	MW3 (0.36), MW1 (0.25), MW2 (0.14)
G12883	Mexico	19.4667	-100.9	MW	MW3 (0.39), MW1 (0.33)
G12884	Mexico	20.5333	-104.8167	MW	MW1 (0.46), MW3 (0.36)
G12890	Mexico	20.3333	-102.2167	MW	MW1 (0.57), MW3 (0.17)
G12893	Mexico	21.2167	-101.8	MW	MW1 (0.63), MW3 (0,23)
G12894	Mexico	20.1333	-102.0833	MW	MW1
G12910	Mexico	20.6167	-101.7167	MW	MW1 (0.52), MW3 (0.44)
G12914	Mexico	20.5333	-103.1833	MW	MW1 (0.42), AW (0.22), MW3 (0.17)
G12916	Mexico	20.6667	-102.45	MW	MW1 (0.60), MW3 (0.20)
G12947	Mexico	20.6667	-102.3833	MW	MW1
G12949	Mexico	20.6667	-102.3833	MW	MW1
G12957	Mexico	20.9	-102.3667	MW	MW1 (0.51), MW3 (0.4)
G12964	Mexico	21.0167	-102.25	MW	MW1
G12986	Mexico	21.6833	-103.1	MW	MW1
G12988	Mexico	20.0833	-104.3667	MW	MW1 (0.60), MW3 (0.34)
G13018	Mexico	18.8833	-99.15	MW	MW3
G13029	Mexico	19.7333	-103.1167	MW	MW3 (0.34), MW1 (0.23), AW (0.20)
G13030	Mexico	19.4667	-103.35	MW	MW3 (0.46), MW1 (0.25)
G15421	Mexico	18.35	-100.2167	MW	MW3
G16796	Argentina	-24.0167	-65.4333	AW	AW
