# Supplementary material for: Spatial and Temporal Scales of Range Expansion in Wild Phaseolus vulgaris
Source: Mol Biol Evol. 2017 Oct 23;35(1):119–31. doi: 10.1093/molbev/msx273 (PMC5850745; doi:10.1093/molbev/msx273)
Supplement: Supplementary Data [file msx273_supp.zip › Table_S2.pdf]

**Table S2:** Results of the accuracy of parameter inferences using cross-validation across difference tolerance rat

| Tolerance level (%) | Founder Population AW | Divergence Time AW | Founder Population PhI | Divergence Time PhI |
|---|---|---|---|---|
| 0.5 | 0.24751822 | 0.06678366 | 0.48354647 | 0.13325946 |
| 1 | 0.27528243 | 0.07637483 | 0.50331527 | 0.14117908 |
| 5 | 0.35918306 | 0.118366 | 0.55657461 | 0.18451144 |

**PhI**, Northern Peru-Ecuador gene pool;**AW**, Andean gene pool
