# Supplementary material for: Spatial and Temporal Scales of Range Expansion in Wild Phaseolus vulgaris
Source: Mol Biol Evol. 2017 Oct 23;35(1):119–31. doi: 10.1093/molbev/msx273 (PMC5850745; doi:10.1093/molbev/msx273)
Supplement: Supplementary Data [file msx273_supp.zip › Table_S3.pdf]

Table S3: List of the *Phaseolus* genotypes sequenced in this study. Passport and sequencing information are shown.

Genotype	Country	Lat	Long	Species	Gene Pool	Barcode	SRA ID	Library Name
E2111	Costa Rica	9.8666	-84.1166	*Phaseolus vulgaris*	MW	ATCCGTCT	SRX2771627	libB
E3121	Costa Rica	10.1666	-84.3833	*Phaseolus vulgaris*	MW	AGTACGCT	SRX2771627	libB
E3132	Costa Rica	10.1666	-84.3833	*Phaseolus vulgaris*	MW	TTCGTTCA	SRX2771627	libB
E3134	Costa Rica	9.8	-84.1166	*Phaseolus vulgaris*	MW	AGGTTCCG	SRX2771627	libB
E3136	Costa Rica	9.85	-84.0666	*Phaseolus vulgaris*	MW	TTCCGGCA	SRX2771627	libB
E3168	Costa Rica	9.65	-83.95	*Phaseolus vulgaris*	MW	CCTGCACT	SRX2771627	libB
E3178	Costa Rica	9.85	-84.0333	*Phaseolus vulgaris*	MW	GAGAATCA	SRX2771627	libB
E3186	Costa Rica	9.7333	-84.0833	*Phaseolus vulgaris*	MW	TTGTCACG	SRX2771627	libB
G10011	Mexico	18.8833	-99.15	*Phaseolus vulgaris*	MW	AACT	SRX2771628	libA
G10018	Mexico	19.6833	-100.9167	*Phaseolus vulgaris*	MW	CCAG	SRX2771628	libA
G10019A	Mexico	19.4667	-100.4833	*Phaseolus vulgaris*	MW	TTGA	SRX2771628	libA
G10256	Portugal			*Phaseolus vulgaris*	D	GAGGTCCG	SRX2771628	libA
G11028	Mexico	24.4667	-104.5833	*Phaseolus vulgaris*	MW	GGTA	SRX2771628	libA
G11050A	Mexico	19.6833	-101.2667	*Phaseolus vulgaris*	MW	ATTG	SRX2771628	libA
G11051	Mexico	20.7667	-103.4	*Phaseolus vulgaris*	MW	CGGT	SRX2771628	libA
G11052	Mexico	20.8	-103.4	*Phaseolus vulgaris*	MW	TGCG	SRX2771628	libA
G11053	Mexico	20.85	-103.2667	*Phaseolus vulgaris*	MW	GTAT	SRX2771628	libA
G11115	Mexico	19.4	-103.4	*Phaseolus vulgaris*	MW	AACCT	SRX2771628	libA
G12851	Guatemala	14.2833	-90.3	*Phaseolus vulgaris*	MW	GTGGT	SRX2771627	libB
G12852	El Salvador	13.9167	-89.85	*Phaseolus vulgaris*	MW	TATAT	SRX2771628	libA
G12856	Peru	-10.3333	-76.1833	*Phaseolus vulgaris*	AW	GGTGTCCA	SRX2771627	libB
G12857	Peru	-11.2167	-75.4833	*Phaseolus vulgaris*	AW	ATCTCGCG	SRX2771628	libA
G12860	Mexico	23.8833	-104.2667	*Phaseolus vulgaris*	MW	GAGCG	SRX2771628	libA
G12861	Mexico	19.4167	-102.0667	*Phaseolus vulgaris*	MW	ACAAT	SRX2771628	libA
G12863	Mexico	20.7833	-104.1833	*Phaseolus vulgaris*	MW	CTCTG	SRX2771628	libA
G12864	Mexico	19.4667	-103.5833	*Phaseolus vulgaris*	MW	TCTGG	SRX2771628	libA
G12865	Mexico	19.3333	-103.25	*Phaseolus vulgaris*	MW	CGGCT	SRX2771628	libA
G12866	Mexico	19.6833	-103.4833	*Phaseolus vulgaris*	MW	GAATT	SRX2771628	libA
G12867	Mexico	21.0833	-104.5	*Phaseolus vulgaris*	MW	ATTAG	SRX2771628	libA
G12868	Mexico	21.3333	-104.5833	*Phaseolus vulgaris*	MW	TGCCG	SRX2771628	libA
G12869	Mexico	19.4167	-102.5833	*Phaseolus vulgaris*	MW	GTGGT	SRX2771628	libA
G12870	Mexico	23.3833	-105.9333	*Phaseolus vulgaris*	MW	ACGTG	SRX2771628	libA
G12872	Mexico	18.9667	-99.1	*Phaseolus vulgaris*	MW	TGAGT	SRX2771628	libA
G12873	Mexico	19	-99.25	*Phaseolus vulgaris*	MW	GCTCT	SRX2771628	libA
G12875	Mexico	17.3167	-96.9	*Phaseolus vulgaris*	MW	CACAA	SRX2771628	libA
G12877	Mexico	18.95	-99.2167	*Phaseolus vulgaris*	MW	GGAAG	SRX2771628	libA
G12878	Mexico	18.35	-99.7667	*Phaseolus vulgaris*	MW	TCCTT	SRX2771628	libA
G12879	Mexico	18.35	-99.9833	*Phaseolus vulgaris*	MW	AATGA	SRX2771628	libA
G12880	Mexico	18.4333	-100.1	*Phaseolus vulgaris*	MW	TCGATCCG	SRX2771627	libB
G12882B	Mexico	18.2833	-100.15	*Phaseolus vulgaris*	MW	CTAGA	SRX2771628	libA
G12883	Mexico	19.4667	-100.9	*Phaseolus vulgaris*	MW	TGGAA	SRX2771628	libA
G12884	Mexico	20.5333	-104.8167	*Phaseolus vulgaris*	MW	TAACA	SRX2771628	libA
G12890	Mexico	20.3333	-102.2167	*Phaseolus vulgaris*	MW	GCCGA	SRX2771628	libA
G12893	Mexico	21.2167	-101.8	*Phaseolus vulgaris*	MW	AGCTA	SRX2771628	libA
G12894	Mexico	20.1333	-102.0833	*Phaseolus vulgaris*	MW	CATACG	SRX2771628	libA
G12898	Mexico	20.1333	-102.0833	*Phaseolus vulgaris*	MW	ATCCGTCT	SRX2771628	libA
G12910	Mexico	20.6167	-101.7167	*Phaseolus vulgaris*	MW	GTACCG	SRX2771628	libA
G12914	Mexico	20.5333	-103.1833	*Phaseolus vulgaris*	MW	AAGTCG	SRX2771628	libA
G12916	Mexico	20.6667	-102.45	*Phaseolus vulgaris*	MW	TCGACG	SRX2771628	libA
G12947	Mexico	20.6667	-102.3833	*Phaseolus vulgaris*	MW	ATCGCG	SRX2771628	libA
G12949	Mexico	20.6667	-102.3833	*Phaseolus vulgaris*	MW	AGTCCG	SRX2771628	libA
G12957	Mexico	20.9	-102.3667	*Phaseolus vulgaris*	MW	CCATCG	SRX2771628	libA
G12964	Mexico	21.0167	-102.25	*Phaseolus vulgaris*	MW	GACACT	SRX2771628	libA
G12986	Mexico	21.6833	-103.1	*Phaseolus vulgaris*	MW	TATCGG	SRX2771628	libA
G12988	Mexico	20.0833	-104.3667	*Phaseolus vulgaris*	MW	CTAGCT	SRX2771628	libA
G13018	Mexico	18.8833	-99.15	*Phaseolus vulgaris*	MW	CGGATG	SRX2771628	libA
G13029	Mexico	19.7333	-103.1167	*Phaseolus vulgaris*	MW	GACTGG	SRX2771628	libA
G13030	Mexico	19.4667	-103.35	*Phaseolus vulgaris*	MW	ACTGCT	SRX2771628	libA
G15421	Mexico	18.35	-100.2167	*Phaseolus vulgaris*	MW	TGACTG	SRX2771628	libA
G16796	Argentina	-24.0167	-65.4333	*Phaseolus vulgaris*	AW	TCGATCCG	SRX2771628	libA
G16798	Argentina	-24.2167	-65.4	*Phaseolus vulgaris*	AW	GGAGAGCT	SRX2771628	libA
G16799	Mexico	18.4333	-100.1667	*Phaseolus vulgaris*	MW	GCGAGT	SRX2771628	libA
G18704	Argentina	-25.9	-65.4833	*Phaseolus vulgaris*	AW	CACCGTCA	SRX2771628	libA
G19833	Peru	-6.2667	-77.75	*Phaseolus vulgaris*	D	TGAGGCCG	SRX2771628	libA
G19833	Peru	-6.2667	-77.75	*Phaseolus vulgaris*	D	ACTTGG	SRX2771627	libB
G19887	Argentina	-24.2	-65.5333	*Phaseolus vulgaris*	AW	TTGTCACG	SRX2771628	libA
G19888	Argentina	-24.1667	-65.6	*Phaseolus vulgaris*	AW	GGTGTCCA	SRX2771628	libA
G19889	Argentina	-24.25	-65.2833	*Phaseolus vulgaris*	AW	AACAAGCT	SRX2771628	libA
G19890	Argentina	-24.5333	-65.35	*Phaseolus vulgaris*	AW	CCATTCCG	SRX2771628	libA
G19891	Argentina	-25.1167	-65.6167	*Phaseolus vulgaris*	AW	TTGCCTCA	SRX2771628	libA
G19892	Argentina	-25.15	-65.65	*Phaseolus vulgaris*	AW	GGTGGACT	SRX2771628	libA
G19893	Argentina	-24.6333	-65.4833	*Phaseolus vulgaris*	AW	AACATTCG	SRX2771628	libA
G19895	Argentina	-26.4333	-65.5167	*Phaseolus vulgaris*	AW	CCACAGCT	SRX2771628	libA
G19896	Argentina	-26.2167	-65.5833	*Phaseolus vulgaris*	AW	TTGTGCCA	SRX2771628	libA

G19897	Argentina	-27.3167	-65.9167	*Phaseolus vulgaris*	AW	AGTGCACG	SRX2771628	libA
G19898	Argentina	-27.3333	-65.95	*Phaseolus vulgaris*	AW	AACAAGCT	SRX2771627	libB
G19901	Argentina	-26.9333	-65.7	*Phaseolus vulgaris*	AW	GAATAGCA	SRX2771628	libA
G19902	Argentina	-26.55	-65.0167	*Phaseolus vulgaris*	AW	CCTACCGT	SRX2771628	libA
G19903	Argentina	-26.3	-65.0333	*Phaseolus vulgaris*	AW	TTCCTATG	SRX2771628	libA
G19906	Guatemala	14.45	-90.7	*Phaseolus vulgaris*	MW	ATGCCT	SRX2771628	libA
G19907	Guatemala	14.45	-90.8167	*Phaseolus vulgaris*	MW	CTCAGG	SRX2771628	libA
G19908	Guatemala	14.5333	-90.8333	*Phaseolus vulgaris*	MW	TAAGCG	SRX2771628	libA
G20559	Costa Rica	9.9333	-84.0833	*Phaseolus vulgaris*	MW	GGAGGTCA	SRX2771628	libA
G21116	Colombia	5.0167	-73.55	*Phaseolus vulgaris*	MW	AGCTTG	SRX2771628	libA
G21118	Colombia	5.0167	-73.55	*Phaseolus vulgaris*	MW	GATCTT	SRX2771628	libA
G21194	Argentina	-24.1167	-65.4167	*Phaseolus vulgaris*	AW	AACT	SRX2771627	libB
G21197	Argentina	-24.05	-65.45	*Phaseolus vulgaris*	AW	CCAG	SRX2771627	libB
G21198	Argentina	-24.0667	-65.3667	*Phaseolus vulgaris*	AW	TTGA	SRX2771627	libB
G21199	Argentina	-23.9167	-65.35	*Phaseolus vulgaris*	AW	GGTA	SRX2771627	libB
G21200	Argentina	-22.25	-65.9333	*Phaseolus vulgaris*	AW	ATTG	SRX2771627	libB
G21201	Argentina	-22.25	-65	*Phaseolus vulgaris*	AW	CGGT	SRX2771627	libB
G21244	Peru	-7.1833	-78.8333	*Phaseolus vulgaris*	PhI	TGCG	SRX2771627	libB
G21245	Peru	-7.1167	-78.7833	*Phaseolus vulgaris*	PhI	GTAT	SRX2771627	libB
G22303	Colombia	4.5333	-73.9167	*Phaseolus vulgaris*	MW	GTAGCGG	SRX2771627	libB
G22304	Colombia	4.4833	-73.9333	*Phaseolus vulgaris*	MW	GCTATG	SRX2771628	libA
G22837	Mexico	26.9333	-106.4167	*Phaseolus vulgaris*	MW	TCAGGT	SRX2771628	libA
G23418	Costa Rica	9.8667	-84.1167	*Phaseolus vulgaris*	MW	TGTACT	SRX2771628	libA
G23419A	Peru	-11.2333	-75.5333	*Phaseolus vulgaris*	AW	CCACG	SRX2771627	libB
G23420	Peru	-11.2	-75.4833	*Phaseolus vulgaris*	AW	TATAT	SRX2771627	libB
G23421	Peru	-12.0167	-74.8833	*Phaseolus vulgaris*	AW	GAGCG	SRX2771627	libB
G23422	Peru	-14	-73.1667	*Phaseolus vulgaris*	AW	ACAAT	SRX2771627	libB
G23423	Peru	-13.85	-72.9667	*Phaseolus vulgaris*	AW	CCATTCCG	SRX2771627	libB
G23424A	Peru	-13.65	-72.9	*Phaseolus vulgaris*	AW	TCTGG	SRX2771627	libB
G23425A	Peru	-13.55	-73.4667	*Phaseolus vulgaris*	AW	CGGCT	SRX2771627	libB
G23426	Peru	-13.6167	-73.2	*Phaseolus vulgaris*	AW	GAATT	SRX2771627	libB
G23427	Peru	-13.6167	-72.8667	*Phaseolus vulgaris*	AW	ATTAG	SRX2771627	libB
G23429	Mexico	18.9667	-98.3833	*Phaseolus vulgaris*	MW	ACGCTG	SRX2771628	libA
G23430	Mexico	17.5667	-99.4833	*Phaseolus vulgaris*	MW	ATGTGCT	SRX2771627	libB
G23431	Mexico	19.0333	-100.05	*Phaseolus vulgaris*	MW	CTGGAG	SRX2771628	libA
G23432	Mexico	18.95	-99.4333	*Phaseolus vulgaris*	MW	CACCTG	SRX2771628	libA
G23435	Guatemala	14.55	-89.6333	*Phaseolus vulgaris*	MW	TGGAGGCG	SRX2771627	libB
G23438	Guatemala	14.6833	-89.8	*Phaseolus vulgaris*	MW	CCGGACT	SRX2771627	libB
G23439	Guatemala	14.4333	-90.1333	*Phaseolus vulgaris*	MW	GTCCAT	SRX2771628	libA
G23440	Guatemala	14.4167	-90.5833	*Phaseolus vulgaris*	MW	TGATCA	SRX2771628	libA
G23441	Guatemala	14.4333	-90.5167	*Phaseolus vulgaris*	MW	CCTAAGCA	SRX2771627	libB
G23442	Bolivia	-17.7833	-65.1667	*Phaseolus vulgaris*	AW	GTGACA	SRX2771628	libA
G23443	Bolivia	-19.2	-64.6	*Phaseolus vulgaris*	AW	ACGTG	SRX2771627	libB
G23444	Bolivia	-19.3	-64.3167	*Phaseolus vulgaris*	AW	TGAGT	SRX2771627	libB
G23445	Bolivia	-21.5333	-64.8667	*Phaseolus vulgaris*	AW	GCTCT	SRX2771627	libB
G23454B	Peru	-13.4667	-72.4167	*Phaseolus vulgaris*	AW	CACAA	SRX2771627	libB
G23455	Peru	-13.5	-72.4833	*Phaseolus vulgaris*	AW	TCCTT	SRX2771627	libB
G23456	Peru	-13.5333	-72.5167	*Phaseolus vulgaris*	AW	AATGA	SRX2771627	libB
G23458	Peru	-13.8167	-71.85	*Phaseolus vulgaris*	AW	ATGCA	SRX2771627	libB
G23459	Peru	-13.5	-72.65	*Phaseolus vulgaris*	AW	CCTTA	SRX2771627	libB
G23462	Colombia	5.0833	-73.6167	*Phaseolus vulgaris*	MW	CTAGA	SRX2771627	libB
G23463	Mexico	28.3333	-108.5	*Phaseolus vulgaris*	MW	CGCAAT	SRX2771628	libA
G23464	Mexico	18.9	-99.0333	*Phaseolus vulgaris*	MW	GCGTAG	SRX2771628	libA
G23469	Mexico	18.9333	-99.1333	*Phaseolus vulgaris*	MW	TAAGCCG	SRX2771628	libA
G23470	Mexico	17.3167	-96.0333	*Phaseolus vulgaris*	MW	ATTCGCG	SRX2771628	libA
G23500	Peru	-13.8667	-72.0667	*Phaseolus vulgaris*	AW	TTGCCTCA	SRX2771627	libB
G23507	Mexico	19.5333	-103.5833	*Phaseolus vulgaris*	MW	GGATCCG	SRX2771628	libA
G23508	Mexico	19.7333	-104.2167	*Phaseolus vulgaris*	MW	CACATCG	SRX2771628	libA
G23511A	Mexico	19.7167	-104.2	*Phaseolus vulgaris*	MW	ATGGCCG	SRX2771628	libA
G23512	Mexico	20.4667	-103.8333	*Phaseolus vulgaris*	MW	TGACGCG	SRX2771628	libA
G23514	Mexico	18.9667	-99.1333	*Phaseolus vulgaris*	MW	GATACCG	SRX2771628	libA
G23520	Mexico	19	-99.0667	*Phaseolus vulgaris*	MW	ACCTTCG	SRX2771628	libA
G23523	Mexico	18.9667	-98.9833	*Phaseolus vulgaris*	MW	TGGAA	SRX2771627	libB
G23526	Mexico	18.9667	-99.0333	*Phaseolus vulgaris*	MW	GGTGGACT	SRX2771627	libB
G23531	Mexico	18.9	-99.9667	*Phaseolus vulgaris*	MW	TATCTCG	SRX2771628	libA
G23535	Mexico	18.95	-99.0833	*Phaseolus vulgaris*	MW	AACATTCG	SRX2771627	libB
G23536	Mexico	18.8167	-98.75	*Phaseolus vulgaris*	MW	GTCAGCG	SRX2771628	libA
G23541	Mexico	18.6667	-99.3167	*Phaseolus vulgaris*	MW	AGTGTCG	SRX2771628	libA
G23545	Mexico	24.3333	-104.2833	*Phaseolus vulgaris*	MW	GAGTTCG	SRX2771628	libA
G23552	Mexico	23.6	-104.3333	*Phaseolus vulgaris*	MW	CCTAGCG	SRX2771628	libA
G23555	Mexico	23.9167	-104.5667	*Phaseolus vulgaris*	MW	CCACTCG	SRX2771628	libA
G23556	Mexico	24	-104.7333	*Phaseolus vulgaris*	MW	TTCTACG	SRX2771628	libA
G23575I	Peru	-13.6333	-72.2167	*Phaseolus vulgaris*	AW	TAACA	SRX2771627	libB
G23579	Ecuador	-3.1833	-79.1667	*Phaseolus vulgaris*	PhI	GCCGA	SRX2771627	libB
G23580C	Ecuador	-3.2	-79.1833	*Phaseolus vulgaris*	PhI	AGCTA	SRX2771627	libB
G23582	Ecuador	-2.2667	-78.9667	*Phaseolus vulgaris*	PhI	CATACG	SRX2771627	libB

G23584	Peru	-5.9333	-79.55	*Phaseolus vulgaris*		PhI	GTACCG	SRX2771627	libB
G23585	Peru	-6.35	-79.4	*Phaseolus vulgaris*		PhI	AAGTCG	SRX2771627	libB
G23587	Peru	-6.35	-79.4	*Phaseolus vulgaris*		PhI	TCGACG	SRX2771627	libB
G23589	Peru	-13.6667	-72.8833	*Phaseolus vulgaris*		AW	ATCGCG	SRX2771627	libB
G23593	Guatemala	14.65	-89.95	*Phaseolus vulgaris*		MW	GAGGCCT	SRX2771628	libA
G23648	Mexico	19.7	-100.55	*Phaseolus vulgaris*		MW	CGTCACG	SRX2771628	libA
G23678	Mexico	20.9	-102.3667	*Phaseolus vulgaris*		MW	ACACGCT	SRX2771628	libA
G23723	Ecuador	-2.2667	-78.9333	*Phaseolus vulgaris*		PhI	CCACAGCT	SRX2771627	libB
G23724	Ecuador	-4.3167	-79.9333	*Phaseolus vulgaris*		PhI	AGTCCG	SRX2771627	libB
G23725	Ecuador	-4.1333	-79.9167	*Phaseolus vulgaris*		PhI	CCTACTCG	SRX2771627	libB
G23726	Ecuador	-1.9667	-78.95	*Phaseolus vulgaris*		PhI	GACACT	SRX2771627	libB
G24318	Argentina	-24.6667	-65.5667	*Phaseolus vulgaris*		AW	TATCGG	SRX2771627	libB
G24322	Argentina	-25.1	-65.6	*Phaseolus vulgaris*		AW	TTGTGCCA	SRX2771627	libB
G24323	Mexico	17.0833	-93.0833	*Phaseolus vulgaris*		MW	AGTGCACG	SRX2771627	libB
G24334A	Mexico	20.0833	-103.85	*Phaseolus vulgaris*		MW	GGAGGCT	SRX2771628	libA
G24338	Mexico	20.1333	-103.85	*Phaseolus vulgaris*		MW	TAGCCTG	SRX2771628	libA
G24340	Mexico	20.1333	-103.9	*Phaseolus vulgaris*		MW	TTAATCG	SRX2771628	libA
G24344	Mexico	20.5833	-103.3333	*Phaseolus vulgaris*		MW	ACCTGTT	SRX2771628	libA
G24345	Mexico	21.0333	-104.4833	*Phaseolus vulgaris*		MW	CGTACGG	SRX2771628	libA
G24348	Mexico	19.6	-103.55	*Phaseolus vulgaris*		MW	GGAGGTCA	SRX2771627	libB
G24350	Mexico	19.6167	-104.2	*Phaseolus vulgaris*		MW	GACCTTG	SRX2771628	libA
G24352	Mexico	20.1	-102.8667	*Phaseolus vulgaris*		MW	ATGTGCT	SRX2771628	libA
G24361	Mexico	19.2833	-103.25	*Phaseolus vulgaris*		MW	GTAGCGG	SRX2771628	libA
G24362	Mexico	19.3	-103.0833	*Phaseolus vulgaris*		MW	TGTAGCT	SRX2771628	libA
G24364	Mexico	19.4	-103.3333	*Phaseolus vulgaris*		MW	CACTATG	SRX2771628	libA
G24365	Mexico	19.4	-103.5333	*Phaseolus vulgaris*		MW	ACTCCGT	SRX2771628	libA
G24368	Mexico	20.4	-102.3333	*Phaseolus vulgaris*		MW	CAGGTGG	SRX2771628	libA
G24375	Mexico	19.15	-100.3333	*Phaseolus vulgaris*		MW	TGACATT	SRX2771628	libA
G24389	Mexico	20.8333	-103.3333	*Phaseolus vulgaris*		MW	GCTTACG	SRX2771628	libA
G24390	Mexico	21.35	-104.5333	*Phaseolus vulgaris*		MW	TTCACTCG	SRX2771628	libA
G24391	Mexico	20.9667	-104.4833	*Phaseolus vulgaris*		MW	GAATAGCA	SRX2771627	libB
G24395	Colombia	4.65	-74.4167	*Phaseolus vulgaris*		MW	CTAGCT	SRX2771627	libB
G24404	Colombia	4.55	-73.9167	*Phaseolus vulgaris*		MW	CGGATG	SRX2771627	libB
G24408	Colombia	4.5667	-73.9167	*Phaseolus vulgaris*		MW	GACTGG	SRX2771627	libB
G24412	Colombia	4.6	-73.9	*Phaseolus vulgaris*		MW	ACTGCT	SRX2771627	libB
G24416	Colombia	4.5	-73.9167	*Phaseolus vulgaris*		MW	TGACTG	SRX2771627	libB
G24423	Colombia	5.0167	-73.55	*Phaseolus vulgaris*		MW	GCGAGT	SRX2771627	libB
G24576	Mexico	16.8	-96.5167	*Phaseolus vulgaris*		MW	AGATGGCG	SRX2771628	libA
G24584	Mexico	16.4667	-92.5333	*Phaseolus vulgaris*		MW	CCTACTCG	SRX2771628	libA
G24589	Mexico	16.25	-91.8333	*Phaseolus vulgaris*		MW	ATCCTCCG	SRX2771628	libA
G24592	Mexico	16.7	-92.85	*Phaseolus vulgaris*		MW	TGGAGGCG	SRX2771628	libA
G24604	Mexico	17.0333	-96.7667	*Phaseolus vulgaris*		MW	GAAGATCG	SRX2771628	libA
G24605	Mexico	20.7	-102.4167	*Phaseolus vulgaris*		MW	CCTCTACG	SRX2771628	libA
G24666	Colombia	4.8	-73.65	*Phaseolus vulgaris*		MW	ATGCCT	SRX2771627	libB
G24671	Colombia	4.8167	-73.6333	*Phaseolus vulgaris*		MW	CTCAGG	SRX2771627	libB
G24690	Colombia	5	-73.4667	*Phaseolus vulgaris*		MW	TAAGCG	SRX2771627	libB
G24693	Colombia	4.9833	-73.4833	*Phaseolus vulgaris*		MW	AGCTTG	SRX2771627	libB
G24752	Colombia	5.0667	-73.4333	*Phaseolus vulgaris*		MW	GATCTT	SRX2771627	libB
G24758	Colombia	5.0833	-73.3833	*Phaseolus vulgaris*		MW	CGATGT	SRX2771627	libB
G24784	Colombia	4.9833	-73.3333	*Phaseolus vulgaris*		MW	GCTATG	SRX2771627	libB
G24798	Colombia	5.1833	-74.1833	*Phaseolus vulgaris*		MW	TCAGGT	SRX2771627	libB
G25228	Mexico	20.9	-105.3833	*Phaseolus lunatus*		W	CACTATG	SRX2771627	libB
G25233	Belize	17.2	-89.1333	*Phaseolus lunatus*		W	AGATGGCG	SRX2771627	libB
G25913		-6.6167	-78.8667	*Phaseolus lunatus*		W	ACTCCGT	SRX2771627	libB
G26721	Ecuador	-3.3333	-79.5667	*Phaseolus lunatus*		W	CAGGTGG	SRX2771627	libB
G2771	Mexico	21.1667	-104.3667	*Phaseolus vulgaris*		MW	GGAAG	SRX2771627	libB
G35758	Guatemala	14.4833	-90.7	*Phaseolus dumosus*		W	TTCACTCG	SRX2771627	libB
G40204	United States	31.8333	-110.7667	*Phaseolus acutifolius*	var. *tenuifolius*	W	TGTAGCT	SRX2771627	libB
G40206	Mexico	25.35	-104.5833	*Phaseolus acutifolius*	var. *acutifolius*	W	TGACATT	SRX2771627	libB
G40756	Bolivia	-17.2167	-66.2	*Phaseolus augusti*		W	GCTTACG	SRX2771627	libB
G40774	Ecuador	-3.2	-79.1833	*Phaseolus augusti*		W	GAGGTCCG	SRX2771627	libB
G50074A	Colombia	5.6833	-75.8667	*Phaseolus vulgaris*		MW	TGTACT	SRX2771627	libB
G50369	Mexico	17.0667	-96.2667	*Phaseolus vulgaris*		MW	GGAGAGCT	SRX2771627	libB
G50384	Guatemala	15.6667	-91.7833	*Phaseolus vulgaris*		MW	GAGAATCA	SRX2771628	libA
G50385	Guatemala	15.65	-91.7	*Phaseolus vulgaris*		MW	CCTGCACT	SRX2771628	libA
G50386	Guatemala	14.7833	-91.4833	*Phaseolus vulgaris*		MW	AGGTTCCG	SRX2771628	libA
G50388	Guatemala	14.75	-91.5167	*Phaseolus vulgaris*		MW	TTCCGGCA	SRX2771628	libA
G50414	Mexico	20.2	-103.8167	*Phaseolus vulgaris*		MW	GAACACCT	SRX2771628	libA
G50503	Guatemala	14.6167	-90.5667	*Phaseolus vulgaris*		MW	CCGTGACG	SRX2771628	libA
G50504	Guatemala	14.7	-90.7833	*Phaseolus vulgaris*		MW	TTCGTTCA	SRX2771628	libA
G50505	Guatemala	14.7167	-90.7833	*Phaseolus vulgaris*		MW	AGTACGCT	SRX2771628	libA
G50506	Guatemala	14.6	-91.1167	*Phaseolus vulgaris*		MW	GAATCACG	SRX2771628	libA
G50507	Guatemala	14.7833	-91.4833	*Phaseolus vulgaris*		MW	CCTAAGCA	SRX2771628	libA
G50518B	Colombia	5.6167	-73.6167	*Phaseolus vulgaris*		MW	ACGCTG	SRX2771627	libB
G50711M	Colombia	6.0833	-75.25	*Phaseolus vulgaris*		MW	ATCTGT	SRX2771627	libB
G50722A	Honduras	13.85	-86.9167	*Phaseolus vulgaris*		MW	CCGGACT	SRX2771628	libA

G50723	Honduras	13.8333	-86.9167	*Phaseolus vulgaris*	MW	CCATCG	SRX2771627	libB
G50724	Honduras	14.1	-86.6833	*Phaseolus vulgaris*	MW	AACCT	SRX2771627	libB
G50725	Honduras	14.0333	-87.05	*Phaseolus vulgaris*	MW	ATCTCGG	SRX2771628	libA
G50797H	Colombia	3.75	-75.4167	*Phaseolus vulgaris*	MW	CTGGAG	SRX2771627	libB
G50859B	Colombia	2.3333	-76.6667	*Phaseolus vulgaris*	MW	CACCTG	SRX2771627	libB
G50879L	Colombia	5.4	-75.5	*Phaseolus vulgaris*	MW	CACCGTCA	SRX2771627	libB
G50898	Costa Rica	9.8167	-84.05	*Phaseolus vulgaris*	MW	TGGACCG	SRX2771628	libA
G51027A	Colombia	5.7167	-75.2833	*Phaseolus vulgaris*	MW	CAGGTT	SRX2771627	libB
G51062	Costa Rica	9.9	-83.9333	*Phaseolus vulgaris*	MW	TCTGAG	SRX2771628	libA
G51282A1	Colombia	6.5	-75.9333	*Phaseolus vulgaris*	MW	GTCCAT	SRX2771627	libB
G51284I	Colombia	6.7833	-75.75	*Phaseolus vulgaris*	MW	TGATCA	SRX2771627	libB
G51361	Argentina	-27.4167	-65.9833	*Phaseolus vulgaris*	AW	TCTGAG	SRX2771627	libB
G51362	Argentina	-27	-65.65	*Phaseolus vulgaris*	AW	GTGACA	SRX2771627	libB
G51363	Argentina	-26.7	-65.4167	*Phaseolus vulgaris*	AW	CGCAAT	SRX2771627	libB
G51364	Argentina	-26.7667	-65.45	*Phaseolus vulgaris*	AW	GCGTAG	SRX2771627	libB
G51367	Argentina	-27.3167	-65.9667	*Phaseolus vulgaris*	AW	TAAGCCG	SRX2771627	libB
G51368	Argentina	-27.3167	-65.9667	*Phaseolus vulgaris*	AW	ATTCGCG	SRX2771627	libB
G51369	Argentina	-27.8333	-65.8167	*Phaseolus vulgaris*	AW	GGATCCG	SRX2771627	libB
G51370	Argentina	-24.65	-65.4333	*Phaseolus vulgaris*	AW	CACATCG	SRX2771627	libB
G51372	Argentina	-24.6167	-65.4167	*Phaseolus vulgaris*	AW	ATGGCCG	SRX2771627	libB
G51373	Argentina	-24.9	-65.4833	*Phaseolus vulgaris*	AW	CCGTGACG	SRX2771627	libB
G51376	Argentina	-24.6667	-65.3667	*Phaseolus vulgaris*	AW	TGAGGCCG	SRX2771627	libB
G51380	Argentina	-24.1167	-65.5333	*Phaseolus vulgaris*	AW	ATCTCGCG	SRX2771627	libB
G51381	Argentina	-24.1333	-65.5333	*Phaseolus vulgaris*	AW	GAAGATCG	SRX2771627	libB
G51658	Costa Rica	9.9	-83.9833	*Phaseolus vulgaris*	MW	CTAGGCG	SRX2771628	libA
PI317349	Mexico	19.43333333	-103.5166667	*Phaseolus vulgaris*	MW	CAGGTT	SRX2771628	libA
PI319441	Mexico	24.23	-104.47	*Phaseolus vulgaris*	MW	CCTACCGT	SRX2771627	libB
PI343950	Guatemala	15.68305556	-91.81694444	*Phaseolus vulgaris*	MW	TTCCTATG	SRX2771627	libB
PI390770	Peru	-13.96694444	-75.08305556	*Phaseolus vulgaris*	AW	CTCTG	SRX2771627	libB
PI417608	Mexico	20.86694444	-102.3669444	*Phaseolus coccineus*	W	ATCCTCCG	SRX2771627	libB
PI417653	Mexico	20.61666667	-101.7166667	*Phaseolus vulgaris*	MW	ACTTGG	SRX2771628	libA
PI430191	Mexico	28.6	-107.1669444	*Phaseolus coccineus*	W	GAACACCT	SRX2771627	libB
PI535413	Mexico	20.08333333	-103.15	*Phaseolus vulgaris*	MW	ATCTGT	SRX2771628	libA
PI535416	Mexico	19.55	-103.6333333	*Phaseolus vulgaris*	MW	CGATGT	SRX2771628	libA
PI535418	Mexico	19.71666667	-104.2	*Phaseolus vulgaris*	MW	ATGCA	SRX2771628	libA
PI535426	Mexico	18.86666667	-98.81666667	*Phaseolus vulgaris*	MW	CCACG	SRX2771628	libA
PI638850	Argentina	-23.48194444	-64.94666667	*Phaseolus vulgaris*	AW	TGACGCG	SRX2771627	libB
PI638864	Argentina	-26.23305556	-65.48305556	*Phaseolus vulgaris*	AW	GATACCG	SRX2771627	libB
PI638865	Argentina	-26.21694444	-65.52666667	*Phaseolus vulgaris*	AW	ACCTTCG	SRX2771627	libB
PI638867	Argentina	-27.79694444	-65.785	*Phaseolus vulgaris*	AW	CTAGGCG	SRX2771627	libB
PI638868	Argentina	-26.38333333	-65.53333333	*Phaseolus vulgaris*	AW	TATCTCG	SRX2771627	libB
PI638869	Argentina	-26.95	-65.7	*Phaseolus vulgaris*	AW	CCACTCG	SRX2771627	libB
PI638870	Argentina	-26.1	-65.6	*Phaseolus vulgaris*	AW	TGGACCG	SRX2771627	libB
PI638871	Argentina	-25.16666667	-65.61666667	*Phaseolus vulgaris*	AW	GTCAGCG	SRX2771627	libB
PI638872	Argentina	-25.16611111	-65.64916667	*Phaseolus vulgaris*	AW	AGTGTCG	SRX2771627	libB
PI638873	Argentina	-24.89611111	-65.80083333	*Phaseolus vulgaris*	AW	GAGTTCG	SRX2771627	libB
PI638874	Argentina	-22.26666667	-64.68333333	*Phaseolus vulgaris*	AW	TGCCG	SRX2771627	libB
PI640964	Argentina	-26.38333333	-65.46666667	*Phaseolus vulgaris*	AW	CCTAGCG	SRX2771627	libB
PI640965	Argentina	-25.16138889	-65.61138889	*Phaseolus vulgaris*	AW	TTCTACG	SRX2771627	libB
PI640966	Argentina	-24.65	-65.36694444	*Phaseolus vulgaris*	AW	GAGGCCT	SRX2771627	libB
PI640967	Argentina	-24.65	-65.36694444	*Phaseolus vulgaris*	AW	CGTCACG	SRX2771627	libB
PI640968	Argentina	-24.71694444	-65.48305556	*Phaseolus vulgaris*	AW	ACACGCT	SRX2771627	libB
PI642122	Argentina	-24.90277778	-65.47527778	*Phaseolus vulgaris*	AW	ATCTCGG	SRX2771627	libB
PI642124	Argentina	-24.67972222	-65.47638889	*Phaseolus vulgaris*	AW	GGAGGCT	SRX2771627	libB
PI653242	Guatemala	14.63333333	-91.11666667	*Phaseolus dumosus*	W	CCTCTACG	SRX2771627	libB
PI661818	Bolivia	-19.53333333	-64.45	*Phaseolus vulgaris*	AW	TAGCCTG	SRX2771627	libB
PI661819	Bolivia	-21.5	-64.15	*Phaseolus vulgaris*	AW	TTAATCG	SRX2771627	libB
W618810	Bolivia	-22.23333333	-64.6	*Phaseolus vulgaris*	AW	ACCTGTT	SRX2771627	libB
W618811	Bolivia	-21.3	-64.85	*Phaseolus vulgaris*	AW	CGTACGG	SRX2771627	libB
WA	Mexico	24.711638	-106.442255	*Phaseolus vulgaris*	MW	GAATCACG	SRX2771627	libB
Blank						CCTTA	SRX2771628	libA
Blank						GACCTTG	SRX2771627	libB
