# Supplementary material for: Spatial and Temporal Scales of Range Expansion in Wild Phaseolus vulgaris
Source: Mol Biol Evol. 2017 Oct 23;35(1):119–31. doi: 10.1093/molbev/msx273 (PMC5850745; doi:10.1093/molbev/msx273)
Supplement: Supplementary Data [file msx273_supp.zip › Table_S4.pdf]

Table S4: Prior distribution of the sampled parameters

Model	Parameter	Distribution	Priors range
Mesoamerican	AW divergence time (T1)	uniform	50000-600000
	PhI divergence time (T2)	uniform	300000-900000
	PhI population size	uniform	100000-300000
	Andean population size	uniform	100000-300000
	Mesoamerican population size	uniform	400000-600000
	North Peru-Ecuador ancestral population size (Np_PhI)	uniform	10-200000
	Andean ancestral population size (Np_AW)	uniform	10-200000
Northern Peru-Ecuador	MW/AW divergence time (T1)	uniform	50000-600000
	MW/AW separation from PhI (T2)	uniform	300000-900000
	North Peru-Ecuador population size (PhI)	uniform	100000-300000
	Andean population size (AW)	uniform	100000-300000
	Mesoamerican population size (MW)	uniform	400000-600000
	Population size of MW/AW separation at T2 (Np)	uniform	10-200000
Protovulgaris	MW/AW divergence time (T1)	uniform	50000-600000
	PhI divergence time (T2)	uniform	300000-900000
	Protovulgaris population size (ANC)	uniform	100000-400000
	PhI population size	uniform	100000-300000
	Andean population size	uniform	100000-300000
	Mesoamerican population size	uniform	400000-600000
	North Peru-Ecuador ancestral population size (Np_PhI)	uniform	10-200000
	Andean ancestral population size (Np_AW)	uniform	10-200000

MW, Mesoamerican wild; **AW**, Andean wild; **PhI**, Northern Peru-Ecuador
